# Ligand Evolution in the Photoactivatable Platinum(IV) Anticancer Prodrugs

**DOI:** 10.3389/fchem.2022.876410

**Published:** 2022-06-09

**Authors:** Jingjing Huang, Weize Ding, Xingfan Zhu, Bingbing Li, Fangang Zeng, Kui Wu, Xiaoqin Wu, Fuyi Wang

**Affiliations:** ^1^ Key Laboratory of Hubei Province for Coal Conversion and New Carbon Materials, School of Chemistry and Chemical Engineering, Wuhan University of Science and Technology, Wuhan, China; ^2^ School of Environment and Natural Resources, Renmin University of China, Beijing, China; ^3^ College of Traditional Chinese Medicine, Shandong University of Traditional Chinese Medicine, Jinan, China; ^4^ Beijing National Laboratory for Molecular Sciences, National Centre for Mass Spectrometry in Beijing, CAS Key Laboratory of Analytical Chemistry for Living Biosystems, Institute of Chemistry, Chinese Academy of Sciences, Beijing, China; ^5^ University of Chinese Academy of Sciences, Beijing, China

**Keywords:** photoactivatable, platinum(IV) prodrug, anticancer, ligand, structure–activity relationship

## Abstract

Photoactivatable Pt(IV) anticancer prodrugs with the structure of [Pt^IV^(N_1_)(N_2_)(L_1_)(L_2_)(A_1_)(A_2_)], where N_1_ and N_2_ are non-leaving nitrogen donor ligands, L_1_ and L_2_ are leaving ligands, and A_1_ and A_2_ are axial ligands, have attracted increasing attention due to their promising photo-cytotoxicity even to cisplatin-resistant cancer cells. These photochemotherapeutic prodrugs have high dark-stability under physiological conditions, while they can be activated by visible light restrained at the disease areas, as a consequence showing higher spatial and temporal controllability and much more safety than conventional chemotherapy. The coordinated ligands to the Pt center have been proved to be pivotal in determining the function and activity of the photoactivatable Pt(IV) prodrugs. In this review, we will focus on the development of the coordinated ligands in such Pt(IV) prodrugs and discuss the effects of diverse ligands on their photochemistry and photoactivity as well as the future evolution directions of the ligands. We hope this review can help to facilitate the design and development of novel photoactivatable Pt(IV) anticancer prodrugs.

## Introduction

**FIGURE 1:**
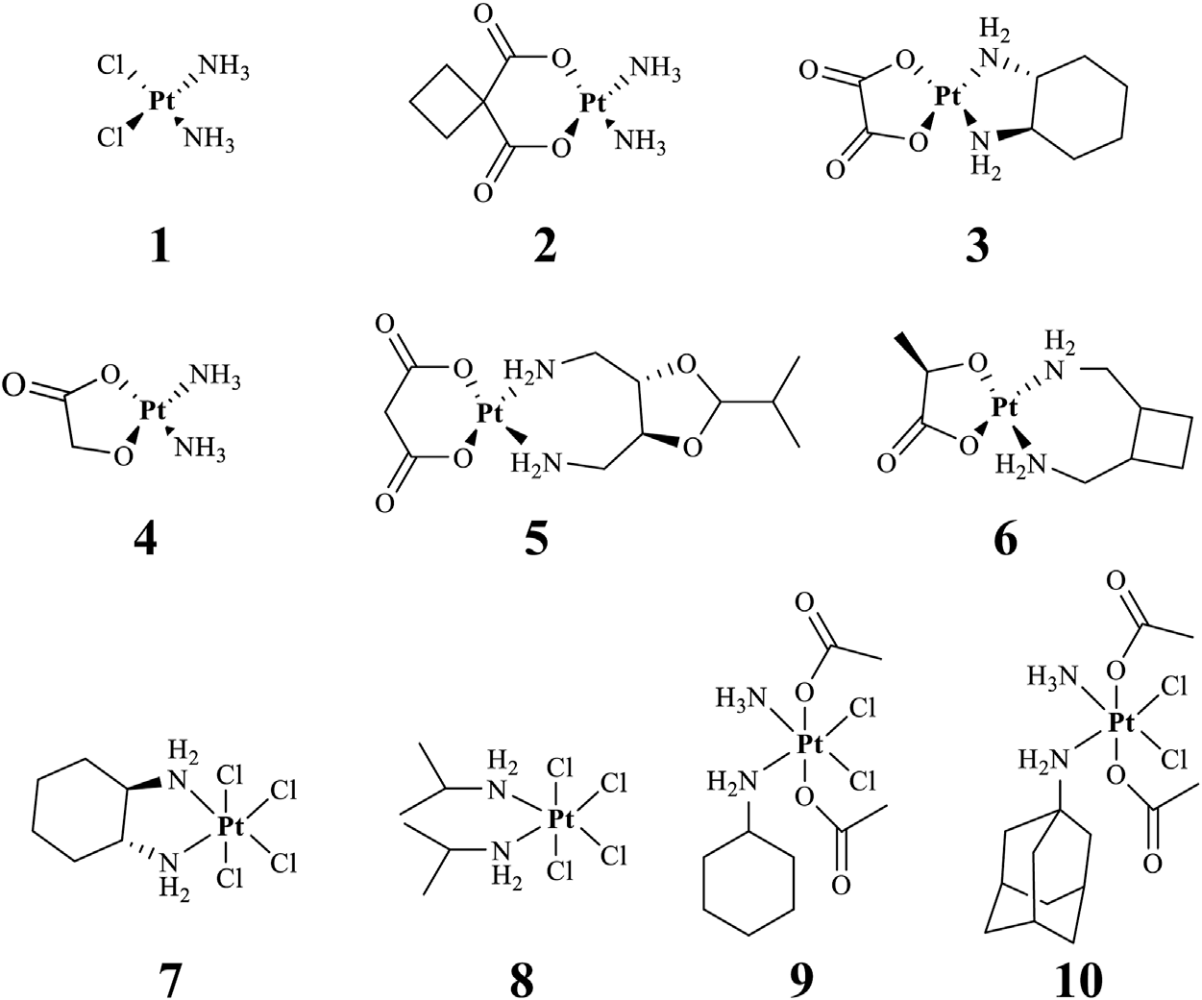
Chemical structures of anticancer Pt(Ⅱ) drugs in clinical use (**1–6**) and Pt(IV) prodrugs entering clinical trials (**7–10)**.

Effective prevention, control, and treatment of cancers have been a persistent public health challenge worldwide and a key to the difficulty of achieving substantial increase in life expectancy (Sung et al., 2021). The lack of effective and selective anticancer agents has existed as a health problem for many years. The indispensability of metals in organisms and their inherent properties are significant contributors to the development of metal antitumor complexes (Frezza et al., 2010; Nafees and Guo, 2014; Eszter Boros and Gasser, 2019). Cisplatin (**1**, Figure 1) is the first metal-based anticancer agent that the FDA approved in the late 1970s for clinical use and is also one of the most widely used anticancer agents (Florea and Busselberg, 2011; Dasari and Tchounwou, 2014; Ghosh, 2019). Later, to improve the shortcomings of cisplatin, such as severe side effects and limited anticancer spectrum, the second- and third-generation platinum drugs carboplatin (**2**) (Mlcouskova et al., 2012) and oxaliplatin (**3**) (Misset et al., 2000) were successively developed and entered clinic use (Figure 1). In contrast to cisplatin, the new generation of platinum drugs has better water solubility and lower toxicities. Other platinum drugs are also approved for clinic use in certain country, for example, nedaplatin (**4**, in Japan in 1995) (Uehara et al., 2011; Shimada et al., 2013), heptaplatin (**5**, in Korea in 1999), and lobaplatin (**6**, in China in 2010) (Boulikas and Vougiouka, 2004) (Table 1).

**TABLE 1:** Clinically approved Pt(II) drugs [adapted from Wheate et al. (2010); Ali et al. (2013); Han et al. (2015); Johnstone et al. (2016); Imran et al. (2018); Ghosh (2019)] and Pt(IV) prodrugs that have entered clinical trials [adapted from Tutsch et al. (1999); Wheate et al. (2010); Imran et al. (2018)].

	Name	Year of approval/phase status	Country/region of approval	Mode of administration	LD_50_ (mg/kg)	Indication	Side effect/limitation
Pt(II) drugs	Cisplatin (also CDDP or Platinol)	1978	Global	Intraperitoneal	12	Testicular, ovarian, bladder, head and neck, and lung cancers	Nephrotoxicity, neurotoxicity, ototoxicity, and acute emesis
	Carboplatin (also JM8 or paraplatin)	1989	Global	Intraperitoneal	120	Ovarian and lung cancer	Myelosuppression, platelet disorders, drug resistance, and peripheral neurotoxicity
	Nedplatin (also 254-S or Aquila	1995	Japan	Intravenous	44.1	Lung, head and neck, and esophageal cancers	Bone marrow suppression and mild nausea
	Heptaplatin (also, SKI 2053R or SunPla)	1999	Korea	Intraperitoneal	196.2	Advanced gastric cancer	Hepatotoxicity and myelosuppression
	Oxaliplatin (also 1-OHP or Eloxatin)	2002	Global	Intraperitoneal	19.8	Colorectal cancer	Neurotoxicity and alimentary canal toxicity
	Lobaplatin (also D-19466)	2010	China	Intraperitoneal	25.7	Testicular, ovarian, and lung cancers	Anemia and leukopenia
Pt(IV) prodrugs	Ormaplatin (or tetraplatin)	Phase I	—	Intraperitoneal and intravenous	23	Breast, ovarian, and myeloma cancers	Too rapid reduction in plasma and severe neurotoxicity
	Iproplatin (or JM9)	Phase III	—	Intraperitoneal	54	Ovary, breast, gastric, pancreatic, and metastatic epidermoid cancer of head and neck	Low cytotoxicity
	Satraplatin (or JM216)	Phase III	—	Oral	30	Ovarian cancer, non-small-cell lung cancer, and small-cell lung cancer	No overall survival benefit
	LA-12	Phase I continued	—	Oral	N.A^a^	Ovary cancer cell lines	N.A.

a not available.

The exploration of “better” Pt(II)-centered drugs is still ongoing (Zhou et al., 2020; Jin et al., 2021), and novel attractive developing directions are also evoked simultaneously by the emergence of Pt(IV) prodrugs. Unlike Pt(II), Pt(IV) has a six-coordination octahedral conformation and is chemically and pharmacologically more inert. While at the tumor microenvironment or inside the cancer cells with the presence of reducing agents, the inert Pt(IV) can be reduced to Pt(II) species to exert anticancer activities. These reduced unexpected reactions take place with off-target biomolecules before Pt(II) binding to DNA in cell nuclei, thereby minimizing side effects (Zhang S. et al., 2020). Several Pt(IV) complexes (**7–10**) have entered clinical trials (Figure 1; Table 1) (Bhargava and Vaishampayan, 2009; Kvardova et al., 2010; Sova et al., 2011; Jelinkova et al., 2014), of which complex **9** (satraplatin) as the first oral Pt(IV) prodrug was demonstrated to be very stable under the highly acidic conditions in the stomach and therefore suitable for oral administration to circumvent the disadvantages of intravenous injection. The metabolism and cytotoxicity of complex **9** depend on cellular GSH levels. However, these Pt(IV) prodrugs have not been approved by the FDA because of their inferior comprehensive performance in clinical practice where these prodrugs still showed similar anticancer mechanisms to traditional Pt(II) drugs, for example, first entering cancer cells and then covalently binding to the DNA. Therefore, switching the activation way of Pt(IV) prodrugs from the GSH-dependent one to new ones, for example, photoactivation, has aroused. Photoactivation of prodrugs can achieve spatially and temporally controlled activation, enabling selective action of the drugs in tumor sites. Three photoactivation strategies, photodynamic therapy (PDT), photothermal therapy (PTT), and photoactivated chemotherapy (PACT), have been developed. PDT requires the simultaneous presence of three essential components: 1) a photosensitizer, 2) oxygen, and 3) light. However, tumor masses are often found under hypoxia conditions, limiting application of conventional PDT (Eales et al., 2016). PTT needs photothermal agents for the conversion of the absorbed light into energy; thus, PTT alone is insufficient to eradicate a tumor and requires combination with other strategies for co-treatment (Ng et al., 2018). In contrast, PACT can produce cytotoxic species by light irradiation in a controlled manner under hypoxia conditions, providing the potential to overcome aforementioned restrictions against PDT and PPT (Imberti et al., 2020).

In recent years, a number of excellent reviews on the advances of photoactivated anticancer Pt(IV) prodrugs have been published. They focused on various aspects of this unique family of prodrugs, for instance, synthesis (Wilson and Lippard, 2014; Xu et al., 2021b), phototherapy potency (Gurruchaga-Pereda et al., 2019; Imberti et al., 2020), photoactivation mode and mechanism of action (Dai and Wang, 2020; Xu et al., 2021b), axial substitutions and modifications, and delivery (Johnstone et al., 2016; Kenny and Marmion, 2019; Ravera et al., 2019; Jia et al., 2021) either in general view of Pt-based anticancer complexes (Imran et al., 2018; Ravera et al., 2022) or in a specific view of a subgroup, for example, diazido-type Pt(IV) prodrugs (Shi et al., 2019; Mu et al., 2021). However, few articles have presented a comprehensive summary and review on the evaluation of the coordinated ligands, namely, non-leaving ligands, leaving ligands, and axial ligands (Wilson and Lippard, 2014), in photoactivated anticancer Pt(IV) prodrugs.

Given that the photoactivation nature of the metal-based complexes largely depends on the structure of coordinated ligands to the metal center, in this review, we focus on evaluation of coordination ligands of the photoactivatable Pt(IV) anticancer prodrugs in the type of [Pt^IV^(N_1_)(N_2_)(L_1_)(L_2_)(A_1_)(A_2_)], where N_1_ and N_2_ are non-leaving ligands, L_1_ and L_2_ are leaving ligands, and A_1_ and A_2_ are axial ligands (Wilson and Lippard, 2014). The non-leaving ligands are usually nitrogen donor, form thermodynamically stable bonds with the platinum center, and often retain in the final platinated adducts of biological molecules. The leaving ligands are generally lost to activate the reduced Pt(II) species to bind to DNA or proteins. They can alter the overall reaction stoichiometry and kinetics of Pt complexes with biological molecules. The axial ligands present only in Pt(IV) complexes can bring versatile characteristics and functions and are usually tumor-targeting moieties, bioactive molecules, nanomaterials, or specific structures to improve physicochemical properties such as solubility, lipophilicity, cellular targeting, and kinetic profiles of the resulting Pt(IV) complexes. They dissociate fast from the Pt(IV) center upon light irradiation, though there is no guarantee that reduction of Pt(IV) will lead to the specific departure of them from the coordination sphere. We aim to provide a systematic review to discuss the effects of diverse ligands on the photochemistry and photoactivity of the Pt(IV) prodrugs as well as the future evolution directions of the ligands. We hope this review would provide new insights into facilitating further design and synthesis of photoactivatable Pt(IV) anticancer prodrugs.

## Leaving Ligand

**FIGURE 2:**
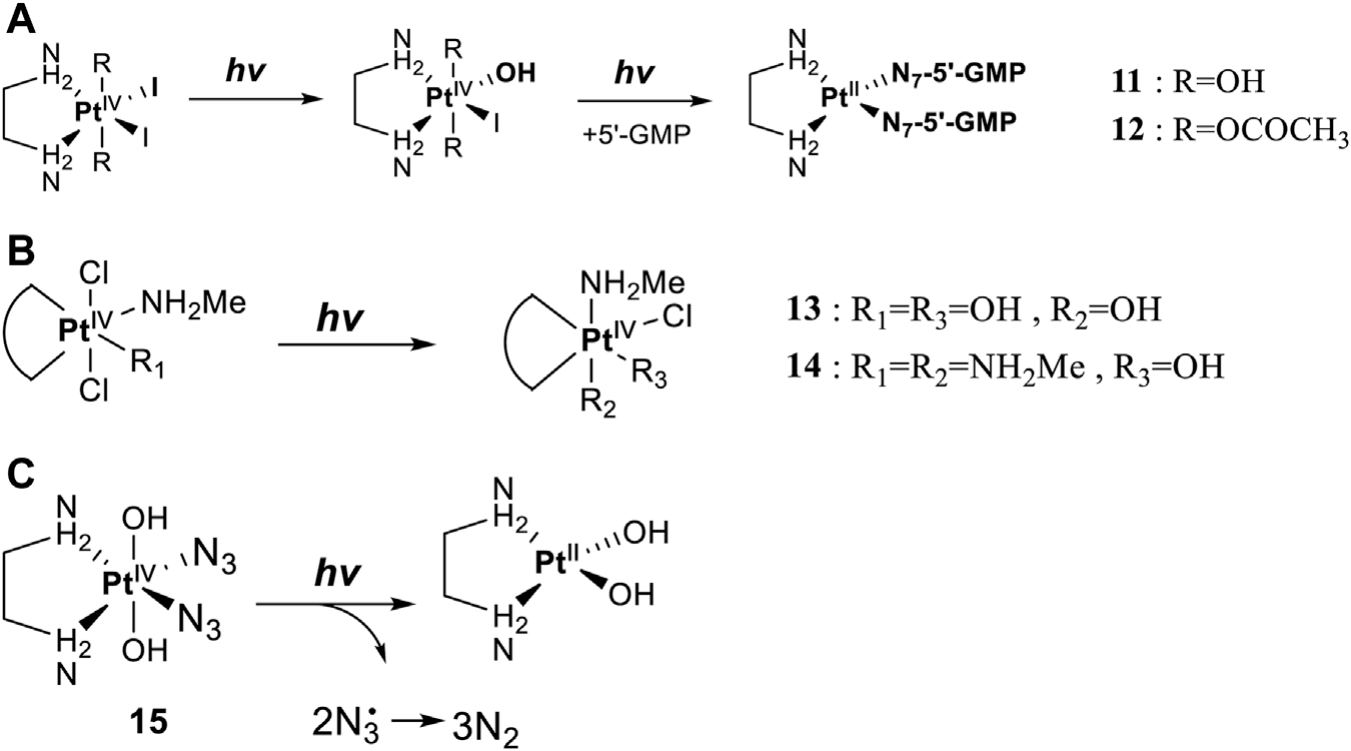
**(A)** Proposed photochemical reaction pathway of **11** and **12** in the presence of 5′-GMP (adapted from Kratochwil et al. (1999)). **(B)** Proposed photo-induced isomerization mechanism of **13** and **14** (adapted from Nakabayashi et al. (2007)). **(C)** Photolysis of complex **15** (adapted from Bednarski et al. (2006); Bednarski et al. (2007)).

Leaving ligands play key roles in photoactive Pt(IV) complexes. Halides are often used as leaving ligands due to easy acquirement and appropriate dissociation inside cells, which facilitates the coordination of reduced products, that is, Pt(II) species to biomolecules after light irradiation. In 1996, the advent of the first generation of photoactive platinum ([Pt^IV^(X)_2_I_2_(en)], X = OH (**11**) or OAc (**12**), en = ethylenediamine) provided a new approach to the reduction of Pt(IV) anticancer complexes (Kratochwil et al., 1996). The energy required to dissociate the diiodide ligands to reach the LMCT-excited state is provided by visible light. Before this activation strategy by light irradiation was reported, the reduction of Pt(IV) mainly relied on biological reductants, such as ascorbic acid and glutathione (GSH) (Khokhar et al., 1993; Ellis et al., 1995), of which the main disadvantage is the uncontrollability of the reduction of Pt(IV). The IC_50_ values of *trans,cis*-[Pt(OH)_2_I_2_(en)] (**11**) inhibiting the growth of the human bladder cancer cell line (TCCSUP) were 7.3 ± 1.6 μM under light (*λ* > 375 nm) irradiation and 9.4 ± 2.2 μM under dark conditions. These values are 11.6 ± 1.7 and 16.5 ± 4.2 µM, respectively, for **12** (Table 2). Although the IC_50_ values in dark and light irradiation for both complexes are close with each other, the antiproliferative activity of **11** indeed increased subjected to light irradiation (Kratochwil et al., 1996). The photochemical reaction pathway of complexes **11** and **12** was studied and elucidated in the presence of mononucleotide guanosine-5′-monophosphate (5′-GMP) (Kratochwil et al., 1999). It was demonstrated that one of the iodine ligands in both complexes first left under light excitation regardless the axial ligands were hydroxyl groups with low spatial resistance or ester groups with high spatial resistance. However, the dissociated products did not readily bind directly to nucleotides at this point because the complexes were still in the octahedral structure. After further light exposure, another iodine ligand and the pair of hydroxyl or ester ligands dissociated, accompanied with the reduction of Pt(IV) to Pt(II), leading to the formation of bis-GMP Pt(II) adducts (Figure 2A). Similar conclusions were obtained by the Bernhard Lippert group in 2007 for complexes **13** and **14** with chloride as the photolytic leaving ligand (Nakabayashi et al., 2007). Surprisingly, however, in addition to hydrolysis during the photolysis process, an unexpected ligand isomerization occurred in **13** and **14**. After one axial chloride and one *cis*-ligand dissociated, the dissociated ligands appeared to return to coordination with Pt but exchanged position with each other (Figure 2B). Such isomerization reached nearly 50% for **13** after 8 h of light irradiation. Similar ligand isomerization of Pt(IV) complexes have also been observed in [Pt(Cl)_2_(NH_3_)_2_(OH)_2_] (Kuroda et al., 1983) and [Pt(NH_3_)_4_(OH)(SO_4_)] (Johnson and Widmer, 1979) but not upon light irradiation. For the former complex, isomerization occurred during recrystallization from water, where the all-*trans* isomer, *trans, trans,trans*-[Pt(Cl)_2_(NH_3_)_2_(OH)_2_], isomerized into the *cis,trans,cis*-[Pt(Cl)_2_(NH_3_)_2_(OH)_2_] isomer, while for the latter, isomerization took placed in the presence of NaOH (pH 12–13) along with the conversion of *trans*-[Pt(NH_3_)_4_(OH)(SO_4_)] into *cis*-[Pt(NH_3_)_4_(OH)_2_]. The mechanism of such isomerization has remained unclear, but the light irradiation cannot be excluded. The catalytic function of traces of Pt(II) may be involved (Kuroda et al., 1983).

**TABLE 2:** IC_50_ values of complexes **11**, **12**, and **15** toward different cell lines under light irradiation or dark condition.

Complex	Cancer cell line	λ_irr_/nm	IC_50_/μM (light)	IC_50_/μM (dark)	Reference
**11**	TCCSUP	375	11.6 ± 1.7	16.5 ± 4.2	Kratochwil et al. (1996b)
**12**	TCCSUP	375	7.3 ± 1.6	9.4 ± 2.2
**15**	5,637	366	63.0 ± 20.2	440 ± 143	Bednarski et al. (2006b)
	5,637/CDDP^a^	—	79.8 ± 16.6	>200	

a CDDP represents cisplatin-resistant 5637 cell line.

Although complex **11** and **12** both showed promising anticancer activities, yet no structures of the photolytic products of them have been reported. The only difference between OAc and OH ligands caused totally distinct interactions of **11** and **12** with calf thymus DNA. No covalent binding of Pt to DNA was measurable after 6 h of incubation in dark, but after light irradiation, 63 ± 13% of the Pt in **12** (*trans,cis*-[Pt(OAc)_2_I_2_(en)]) was detected to bind to DNA. In contrast, only 10 ± 2% of the Pt in **11** (*trans,cis*-[Pt(OH)_2_I_2_(en)]) was found to bind to DNA after 6 h of illumination, while a marked increase in DNA platination took place after addition of GSH (Kratochwil et al., 1996). These comparable results imply that the photoreduction of Pt(IV) to Pt(II) species occurred upon the photoactivation of **12,** leading to DNA platination, but for the photolysis of the *trans*-dihydroxo analog **11**, light probably activated only the substitution of the iodide ligands by water rather than a reduction of Pt(IV) to Pt(II) (Kratochwil et al., 1996). This indicated that complex **11** might be not sensitive to light and needed GSH to reduce Pt(IV) to Pt(II), triggering DNA platination.

Bednarski et al. (2007) carried out an electronic absorption spectroscopic study on halogen-substituted *cis,trans,cis*-[Pt^IV^(X)_2_(Cl)_2_(NH_3_)_2_] (X = Cl, Br or I) with water as solvent. It was found that from chloride to bromide and iodide, the energy absorbed by ligand-to-metal charge transfer (LMCT) decreased sequentially with the decrease of electronegativity of the ligand. As a consequence, the iodide Pt(IV) prodrug required the lowest energy of light to initiate the photochemical process accompanied with the photochemical reduction of Pt(IV) to Pt(II).

However, although iodine being the leaving ligand of Pt(IV) prodrugs that has been studied for decades, there has not been much progress in the controlled photochemical reduction of the Pt(IV) center (Štarha et al., 2019) because biological reductants such as ascorbic acids and glutathione could reduce the coordinated Pt(IV) to Pt(II) under dark conditions. This consequently leads to an apparent dark cytotoxicity which always is a major problem for clinical application of Pt(IV) prodrugs (Kratochwil and Bednarski, 1999a). On the other hand, using halides as leaving ligands requires short wavelength of light to initiate photolysis, which always means poor tissue penetration. Therefore, halides are not ideal leaving ligands for photoactivated Pt(IV) prodrugs.

To this end, the azide group as a prominent pseudo-halogen was brought into thought (Portius and Davis, 2013). The azide as the leaving group in Pt(IV) prodrugs was first proposed by Vogler et al. (1978b). It was reported that the complex has an (N_3_→Pt) CT leap at λ = 302 nm. The photochemical reduction at this wavelength forms two azide radicals; meanwhile, Pt(IV) is reduced to Pt(II) without the formation of Pt(III) species:

$${\left[{\text{Pt}}^{\text{IV}}{\left(\text{CN}\right)}_{4}{\left({\text{N}}_{3}\right)}_{2}\right]}^{2-}\to {\left[{\text{Pt}}^{\text{II}}{\left(\text{CN}\right)}_{4}\right]}^{2-}+2{\text{N}}_{3}^{•}$$

Then, the photoreactions of *trans*-[Pt^IV^(CN)_4_(N_3_)(X)] and *trans*-[Pt^IV^(CN)_4_(X)_2_](X = Cl or Br) were comparatively studied by Vogler et al. (1978a). On the one hand, *trans*-[Pt^IV^(CN)_4_(X)_2_] could be regenerated in aqueous solution due to the stability of the photoreduction product [Pt^II^(CN)_4_]^2−^ in case the resulting halogen radicals were not scavenged. On the other hand, the presence of the azide group significantly increased the irradiation wavelength of *trans*-[Pt^IV^(CN)_4_(N_3_)(X)] compared to *trans*-[Pt(CN)_4_(X)_2_]. The quantum yield to produce [Pt^II^(CN)_4_]^2−^ from azide Pt(IV) prodrugs was also promoted, attributable to the instability of the photo-generated azide radicals (Table 3). Collectively, the introduction of azide was apparently beneficial for photoactivation of Pt(IV) prodrugs, though the later studies from the same group showed that the maximum wavelength (305 nm) of LMCT band of the [Pt(N_3_)_6_] moiety did not change significantly compared to that (300 nm) of [Pt(CN)_4_(N_3_)_2_] (Vogler et al., 1988). Despite no photocytotoxicity study on this kind of cyano Pt(IV) prodrugs was presented at that time, Vogler’s work greatly contributed to the development of the second generation of photoactive azide-based Pt(IV) prodrugs, for example, *cis,trans*-[Pt(en)(N_3_)_2_(OH)_2_] (**15**) in 2003 reported by Brabec and Sadler et al. (Figure 2C) (Kašpárková et al., 2003). After light irradiation at 366 nm, the IC_50_ value (63.0 ± 20.2 μM) of complex **15** toward the human bladder cancer cell line 5637 was nearly 6-fold lower than that in the dark conditions (440 ± 143 μM) (Bednarski et al., 2006). Considering the similar activities of complexes **11** and **12** under light irradiation and dark conditions, the promotion of antiproliferative activity of **15** subjected to light exposure indicates the much higher stability of azide Pt(IV) prodrugs over iodide Pt(IV) prodrugs under dark conditions (Table 2). Moreover, even to the cisplatin-resistant 5637 cells, the photocytotoxicity of complex **15** maintained at a similar level to the sensitive counterpart, showing the higher developing potentials of the azido Pt(IV) prodrugs. *In vitro* study observed a terminated RNA synthesis on a 212-bp fragment of pSP73 KB DNA by complex **15** after irradiation, and the major stop sites appeared mainly at GG sequences, which were similar to those produced by cisplatin (Kasparkova et al., 2003). Later two-dimensional [^1^H,^15^N] NMR studies further confirmed such preferential binding sequence context, but the platination rate by complex **15** was shown to be faster than that by cisplatin (Bednarski et al., 2006). Combining with the dramatic effects on the morphology of cancer cells including disintegration of nuclei by complex **15**, these results suggest a different mechanism of action of **15** from cisplatin.

**TABLE 3:** Quantum yields of the cyano fragment [Pt(CN)_4_]^2−^ derived from [Pt(CN)_4_(X)(Y)] under light irradiation at various wavelength [adapted from Vogler et al. (1978a)].

	[Pt(CN)_4_(N_3_)(Cl)]		[Pt(CN)_4_(N_3_)(Br)]		[Pt(CN)_4_(Cl)_2_]	[Pt(CN)_4_(Br)_2_]
Solvent	Ethanol	Water	Ethanol	Water	Ethanol	Ethanol
Irradiation wavelength	300 nm	300 nm	300 nm	300 nm	254 nm	254 nm
Quantum yield	0.35 ± 0.02	0.22 ± 0.02	42 ± 0.02	0.26 ± 0.02	34 ± 0.02	25 ± 0.03

Pt(IV) prodrugs with azide as the leaving ligand have become a breakthrough to develop the photoactivatable platinum-based antitumor prodrugs. The leaving azido ligand plays a vital role in the changes in the wavelength of LMCT bands of the resulting complexes as the difference of optical electronegativity between the ligand and the metal center determines the energy of LMCT (Knoll et al., 1990; Imran et al., 2018). The azido Pt(IV) prodrugs have a small LMCT (N_3_→Pt) transition energy, making them more readily to be reduced during light exposure, compared to the highly stable halido Pt(IV) complexes. The presence of azido ligand with its strong electron-donating nature also reduces the reactivity of Pt(IV) with biological reductants in dark, making the photolysis of the azide Pt(IV) complexes more controllable than that of the iodide Pt(IV) prodrugs, of which the phototoxicity and dark toxicity to certain cancer cells have no obvious difference (Table 2). It is notable that the halide ligands in the Pt(IV) prodrugs can be oxidized to form stable halogen radicals, and the steric configuration would isomerize during photolysis. Meanwhile, the photolysis of azide Pt(IV) prodrugs generated highly unstable azide radicals or N_2_, thus preventing rearrangement of the ligands and increasing the content of the reduced Pt(II) species binding to the DNA target.

## Non-Leaving Ligand

Amine is usually used as the non-leaving ligands in both Pt(II) anticancer drugs and Pt(IV) prodrugs. Sadler and co-workers conducted in-depth studies on the relationships between non-leaving ligands and the biological activity of photoactivatable diazido Pt(IV) prodrugs. In 2003, they demonstrated that under UV irradiation (366/355 nm), both complex **16** *cis,trans,cis*-[Pt^IV^(en)(N_3_)_2_(OH)_2_] with a chelating amine ligand and complex **17** *cis,trans,cis*-[Pt^IV^(N_3_)_2_(OH)_2_(NH_3_)_2_] with non-chelating ammonia ligands (Table 4) had good photocytotoxicity toward both 5637 and cisplatin-resistant 5637 cancer cell lines with **17** achieving lower IC_50_ values than **16** (Müller et al., 2003). In the dark, no reactions of complexes **16** and **17** with 5′-GMP and d (GpG) in human blood plasma were observed, but a slow reaction of both the complexes with GSH over a period of several weeks was observed. Such promising stability under physiological conditions is very vital as it has been acknowledged as one of the important factors determining the success of photochemotherapeutic agents. 1D ^1^H, 2D [^1^H,^15^N] HSQC, and 2D [^1^H,^15^N] HSQC-TOCSY NMR spectroscopic results revealed the formation of bis-GMP adducts [Pt(en)(GMP-N7)_2_]^2+^ and [Pt(NH_3_)_2_(GMP-N7)_2_]^2+^ when complexes **16** and **17** were individually incubated with 2 mol. equiv. of 5′-GMP subjected to photoactivation. Moreover, crosslinking adducts, [Pt(en){d(GpG)-N7^1^,N7^2^}]^2+^ and [Pt(NH_3_)_2_{d(GpG)-N7^1^,N7^2^}]^2+^, were also detected when these two complexes individually reacted with 1 mol. equiv. of d (GpG) under irradiation. The formation of these bifunctional GG adducts with **16** or **17** is similar to that with cisplatin, but the rates with the photoactivated **16** and **17** are faster than with cisplatin. However, no oxidation on the bases has been observed in this work (Müller et al., 2003). The reason may be ascribed to the low sensitivity of the NMR technique which could not detect low abundance of base oxidative adducts in these cases.

**TABLE 4:** Chemical structures of complexes **16–24** and IC_50_ of complexes toward different cancer cell lines.

	Complex	R_1_	R_2_	λmax/nm of the LMCT band (N3→Pt)	λ_irr_/nm	Cell line	IC_50_ (light)/μM	IC_50_ (dark)/μM	PI IC_50_ (dark)/IC_50_ (light)	Reference
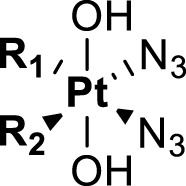	**16**	NH_2_CH_2_CH_2_NH_2_		315	366/365	5637	63.0 ± 20.2	440 ± 143	7.0	Bednarski et al., (2007); Farrer et al., (2010a)
						5637-CDDP	79.8 ± 16.6	>200	>2.5	
						A2780	16 ± 1	67 ± 8	4.2	
						A2780cis	22 ± 3	82 ± 8	3.7	
	**17**	NH_3_	NH_3_	256	366	5637	49.3 ± 28.1	357 ± 81	7.2	Bednarski et al., (2007); Bednarski et al., (2006); Farrer et al., (2010a)
					—	5637-CDDP	79.8 ± 16.6	>200	2.5	
					365	HaCaT	169.3	>287.9	>1.7	
					—	A2780	135.1	287.9	2.1	
					—	A2780cis	204.9	287.9	1.4	
	**21**	NH_3_	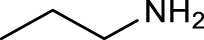	257	365	HaCaT	203.9	>256.9	>1.3	—
						A2780	93.4	>256.9	>2.8	
	**22**	NH_3_	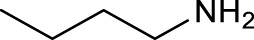	258	365	HaCaT	149.9	>247.9	>1.7	Farrer et al. (2010a)
	**23**	NH_3_	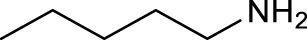	258	365	HaCaT	224.3	>239.6	>1.1	—
						A2780	>239.6	>239.6	—	
						A2780cis	>239.6	>239.6	—	
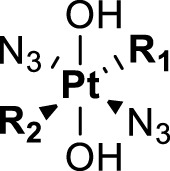	**18**	NH_3_	NH_3_	285	365	HaCaT	121.2	>287.9	>2.4	Farrer et al. (2010a)
						A2780	99.2	>287.9	>2.9	
						A2780cis	163.6	>287.9	>1.8	
	**19**	NH_3_	MeNH_2_	286	420	HaCaT	163.3	>276.8	>1.7	Farrer et al. (2010a); Mackay et al. (2009b)
					365	HaCaT	65.5	>276.8	>4.2	
					—	A2780	39.8	>276.8	>7.0	
					—	A2780cis	128.7	>276.8	>2.2	
	**20**	NH_3_	EtNH_2_	285	420	HaCaT	98.3	>266.5	>2.7	—
					365	HaCaT	68.3	>266.5	>3.9	
					—	A2780	58.4	>266.5	>4.6	
					—	A2780cis	90.1	>266.5	>3.0	Westendorf et al. (2011)
	**24**	NH_3_	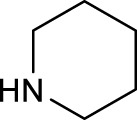	287.5	366	5637	29.17 ± 2.23	>80	>2.7	

The bold values represent the numbering of the complexes.

Further *in vitro* cellular experiments confirmed that **17** caused more severe damages to cancer cells, evidenced by significant changes in cell morphology in response to light irradiation. However, in contrast to the better activity of cisplatin over transplatin, complex **17** with *cis*-configuration showed less cytotoxicity than complex **18** with *trans*-configuration to HaCaT, A2780, and A2780cis cancer cell lines under 365-nm light irradiation (Mackay et al., 2006). This suggests that the *trans*-isomers are more favorable for this kind of diazido photoactive Pt(IV) prodrugs, though both of the *trans*- and *cis*-Pt(IV) showed good dark stability. This was confirmed by further studies on aliphatic amine ligands with different chain lengths. Complexes **19** and **20** prepared by replacing NH_3_ with MeNH_2_ and EtNH_2_ in *trans*-Pt(IV) complexes exhibited better photocytotoxicity than complex **17** to various cancer cell lines subjected to irradiation of 365 nm light (Mackay et al., 2009a; Farrer et al., 2010a). However, no obvious increasing activity correlated well with the elevated number of carbon chain from NH_3_ to MeNH_2_ and EtNH_2_ in complexes **18**, **19**, and **20**, respectively, when using 365-nm light to irradiate. Similar not-well-correlation result was also observed for *cis*-Pt(IV) complexes **21**, **22,** and **23**, in which the non-leaving NH_3_ ligand was replaced by CH_3_(CH_2_)_2_NH_2_, CH_3_(CH_2_)_3_NH_2_, and CH_3_(CH_2_)_4_NH_2_, respectively, under the same photoactivation condition (Farrer et al., 2010a). While under 420 nm light irradiation, the IC_50_ values of complex **19** with MeNH_2_ as non-leaving ligand to HaCaT cancer cells significantly reduced, compared to that of complex **20** with EtNH_2_ as non-leaving ligand. Such correlation between cytotoxicity under different exciting wavelengths and the chain length of aliphatic amines may be due to the potential effects of higher light energy on cancer cells. Moreover, complex **24** prepared by replacing NH_3_ with the cyclic amine ligand, piperidine, also showed better activity toward the 5637 cell line (Westendorf et al., 2011). A detailed comparison for the photocytotoxicity of complexes **16**–**24** is given in Table 4.

The development of diazido Pt(IV) anticancer prodrugs in Sadler’s group achieved a further step by substituting amine with the nitrogen heterocyclic ring, for example, pyridine, thiazole, or imidazole, as the non-leaving ligands in *cis*- or *trans*-position (complexes **25**–**43**; Table 5) (Farrer et al., 2010a; Farrer et al., 2010b). Larger arene ligands have several advantages over NH_3_ groups: i) the bulkier ring can prevent off-target interactions of the Pt(IV) complexes as well as their photoproducts; ii) they can promote π-stacking and intercalation ability of the Pt(IV) complexes with DNA, favoring the binding to the DNA target; and iii) they can produce a bathochromic shift in the absorption spectrum closing the clinical needs (Gurruchaga-Pereda et al., 2019). Such modification nearly makes all this series of complexes activatable by the visible blue light at 420 nm. In particular, complex **36** can be activated by green light at 465 nm. Under such a low energy irradiation at 465 nm, complex **36** still showed promising cytotoxicity to the A2780 cancer cell line with a IC_50_ value being 7.1 ± 0.4 μM, while almost no dark cytotoxicity (IC_50_ in dark >100 μM) and no obvious damage to normal cells (MRC5) were observed (Table 5). Nevertheless, the higher energy irradiation at 365 nm endowed complex **36** higher activity with IC_50_ value as 1.4 μM to both HaCaT and A2780 cancer cell lines. Meanwhile, high inertness under dark conditions was also confirmed by the PI value (Dark IC_50_ value/Light IC_50_ value) over 151. Our research revealed that such promising anticancer activities of the azido Pt(IV) prodrugs are highly correlated with the synergetic effects of the covalent binding of reduced Pt(II) species and the generated reactive oxygen and nitrogen species like hydroxyl radicals, azidyl radicals, and singlet oxygen from their photolysis, to biological molecules. For example, using high resolution mass spectrometry analysis, we demonstrated that complex **36** upon blue light irradiation produced Pt(II) species and highly reactive radicals to covalently bind to and induce oxidation at all the four nucleobases (Zhang J. et al., 2020; Cheng et al., 2020; Liang et al., 2020). The binding preference of complex **36** to the four nucleobases was in the order as: G > A and C > T, which is totally distinct to that of cisplatin. Moreover, guanine, adenine, and cytosine can be di-platinated by **36** to saturate all potential binding sites. The induced oxidation of adenine, cytosine, and thymine bases by **36** was also first reported by us. The representative oxidation adducts were 8-hydroxyl-adenine (8-OH-A), 2,8-diOH-adenine (2,8-diOH-A), and 4,6-diamino-5-formamido-adenine (FapyA) for adenine; 5-hydroxycytosine (5-OH-C), 5,6-dihydroxy-5,6-dihydrocytosine (cytosine glycols, C-Gly), and 6-hydroxyl-5,6-dihydrocytosine (cytosine photohydrate, C-PH) for cytosine; and *cis-* and *trans*-diastereomers of 5,6-dihydroxy-5,6-dihydrothymidine (ThdGly), 5-formyl-2′-deoxyuridine (FormdUrd), and 5-hydroxymethyl-2′-deoxyuridine (HMdUrd) for thymine. Meanwhile for guanine, apart from 8-hydroxyguanine (8-OH-G) and N-formylamidoiminohydantoin (RedSp), more oxidation adducts were discovered in our studies, such as dehydroguanidinohydantoin (DGh), spiroiminodihydantoin (Sp), and 2,6-diamino-4-hydroxy-5-formamidopyrimidine (FapyG). The mass spectrometry analysis has high sensitivity, enabling discovery and identification of low abundant and diverse oxidative species of nuclear bases induced by Pt(IV) complexes under light irradiation and benefiting for better understanding in the uncovered dual-action feature of these highly potent photo-antiproliferative Pt(IV) prodrugs. The similar covalent binding and induced oxidation were also found when complex **36** reacted with peptides/proteins such as substance P, [Lys]^3^-bombesin, and thioredoxin under visible light (Du et al., 2018; Wootton et al., 2018). Interestingly, the reduced Pt(II) species from **36** was found to covalently bind to histidine, glutamic acid, glutamine, and methionine residues, and the oxidation induced by generated ROS was detected on tryptophan additional to methionine and cysteine (Du et al., 2018). These collectively indicate that the introduction of pyridine as non-leaving ligands into photoactivatable Pt(IV) prodrugs significantly enhances its photobiological activities, endowing the resulting complexes’ distinct mechanism of action from conventional Pt(II) anticancer drugs.

**TABLE 5:** Chemical structures of complexes **25–43** and IC_50_ values of complexes toward different cancer cell lines.

	Complex	R_1_	R_2_	λ_max_/nm of the LMCT band (N_3_→Pt)	λ_irr_/nm	Cell line	IC_50_ (light)/μM	IC_50_ (dark)/μM	PI IC_50_ (dark)/IC_50_ (light)	References
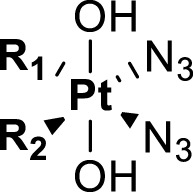	**25**	NH_3_	Py	258	365	HaCaT	100.9	>244.4	>2.4	Farrer et al. (2010a)
					420		116.5	>244.4	>2.1	
					365	A2780	79.6	>244.4	>3.1	
					365	A2780cis	108.7	>244.4	>2.3	
	**27**	NH_3_	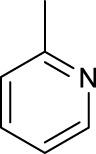	258	365	HaCaT	131.0	>236.3	>1.8	Farrer et al. (2010a)
					420		89.9	>236.3	>2.6	
					365	A2780	65.9	>236.3	>3.6	
						A2780cis	165.2	>236.3	>1.4	
	**29**	NH_3_	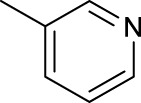	259	365	HaCaT	>236.2	>236.2	—	Farrer et al. (2010a)
						A2780	63.6	>236.2	>3.7	
						A2780cis	>236.2	>236.2	—	
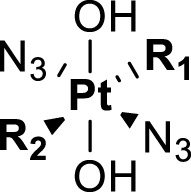	**26**	NH_3_	Py	289	365	HaCaT	6.8	>244.4	>35.9	Farrer et al., (2010a); Pizarro et al., (2014)
					420	—	85.9	>244.4	>2.8	
					365	A2780	1.9	>244.4	>128.6	
					420	—	25.4	>244.4	>9.6	
					365	A2780cis	16.9	>244.4	>14.5	
					365	OE19	10.0	>244.4	>24.4	
					420	—	32.0	>244.4	>7.6	
	**28**	NH_3_	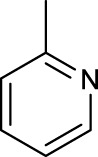	292	365	HaCaT	54.0	>236.3	>4.4	Farrer et al., (2010a); Mackay et al., (2009b); Wootton et al., (2018)
						A2780	51.0	>236.3	>4.6	
						A2780cis	59.7	>236.3	>4.0	
	**30**	NH_3_	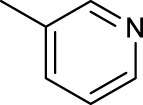	289	365	HaCaT	22.0	144.1	6.6	Wootton et al. (2018)
					420	—	14.6	144.1	9.9	
					365	A2780	2.6	26.8	10.3	
					—	A2780cis	2.9	57.7	19.9	
	**31**	NH_3_	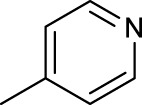	289	365	HaCaT	7.1	97.8	13.8	Farrer et al., (2010a); Mackay et al., (2009a)
					420	—	11.9	97.8	8.2	
					365	A2780	4.2	108.7	25.9	
					—	A2780cis	5.4	134.9	25.0	
	**32**	NH_3_	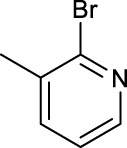	287	365	HaCaT	61.0	108.0	1.8	Farrer et al. (2010a)
						A2780	15.8	31.3	2.0	
						A2780cis	38.2	54.4	1.4	
	**33**	NH_3_	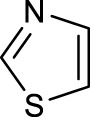	289	365	HaCaT	4.5	>241.0	>53.6	Mackay et al., (2009b); Zhao et al. (2013)
					420	HaCaT	19.8	>241.0	>12.2	
					365	A2780	5.5	186.9	34.0	
					—	A2780cis	9.9	>241.0	>24.3	
	**34**	MeNH_2_	Py	289	365	HaCaT	2.6	>236.3	>90.8
					420	—	14.7	>236.3	>16.0
					365	A2780	2.3	>236.3	>102.7
					420	—	6.6	>236.3	>35.8
					365	A2780cis	4.4	>236.3	>53.7
					420	—	13.2	>236.3	>17.9
					365	OE19	10.1	>236.3	>23.4
					420	—	13.9	>236.3	>17.0
	**35**	MeNH_2_	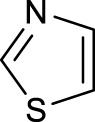	289	365	HaCaT	3.5	>232.9	>66.5	—
					420		11.2	>232.9	>20.7	
					365	A2780	3.2	>232.9	>72.8	
					420		28.2	>232.9	>8.3	
					365	A2780cis	5.3	>232.9	>43.9	
					420		6.4	>232.9	>36.4	
					365	OE19	6.2	>232.9	>37.6	
					420		19.3	>232.9	>12.1	
	**36**	Py	Py	294	365	HaCaT	1.4	>212.3	>151.6	Farrer et al., (2010b); Farrer et al., (2010a); Zhao et al., (2013)
					420	—	9.5	>212.3	>22.3	
					365	A2780	1.4	>212.3	>151.6	
					420	—	8.3	>212.3	>25.6	
					365	A2780cis	14.5	>212.3	>14.6	
					365	OE19	4.7	>212.3	>25	
					420	—	8.4	>212.3	>45	
					365	HepG2	2.5	>212.3	>85	
					465	A2780	7.1 ± 0.4	>100	>14.1	
					—	A549	51.9 ± 2.5	>100	>1.9	
					—	PC3	55.6 ± 0.9	>100	>1.8	
					—	MRC5	—	>100	—	
	**37**	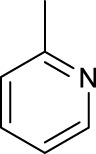	Py	292	420	A2780	14.5	105.9	7.3	Mackay et al. (2009b)
						A2780cis	15.5	82.2	5.3	
	**38**	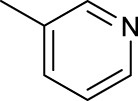	Py	294	420	A2780	4.0	>206.0	>51.5	—
						A2780cis	3.3	>234.3	>71.0	
						OE19	5.5	>209.0	>38	
	**39**	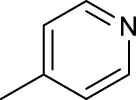	Py	294	420	A2780	5.4	>205.2	>38	—
						A2780cis	4.6	>207.0	>45	
						OE19	12.3	>209.1	>17	
	**40**	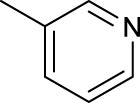	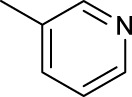	295	420	A2780	7.2	>200.2	>27.8	—
						A2780cis	10.4	>200.7	>19.3	—
	**41**	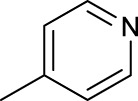	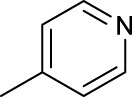	293	420	A2780	2.1	>199.5	>95	—
						A2780cis	4.1	>200.1	>48.8	
						OE19	8.2	>196.8	>24	
	**42**	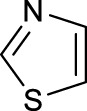	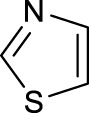	295	420	A2780	2.4	115.2	48	—
						A2780cis	2.9	>205.9	>71.0	
						OE19	7.6	>205.2	>27	
	**43**	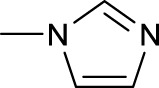	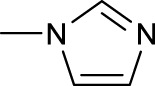	290	420	A2780	56.3	>208.3	>3.7	—
						A2780cis	164.2	>213.5	>1.3	
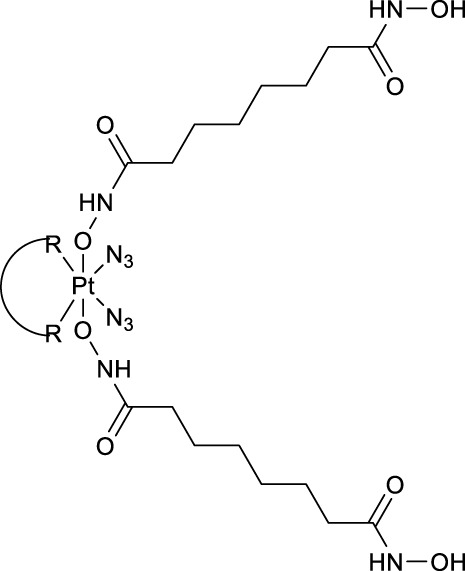	**44**	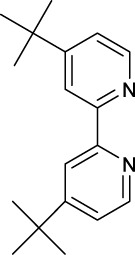	—	318	365	A2780	3.3 ± 0.3	57 ± 6	17.3	Kasparkova et al. (2015)
						A2780cis	3.9 ± 0.5	18 ± 5	4.6	
					420	A2780	8.4 ± 0.4	31 ± 2	3.7	
						A2780cis	10.2 ± 0.2	24.2 ± 0.7	2.4	

The bold values represent the numbering of the complexes.

**FIGURE 3:**
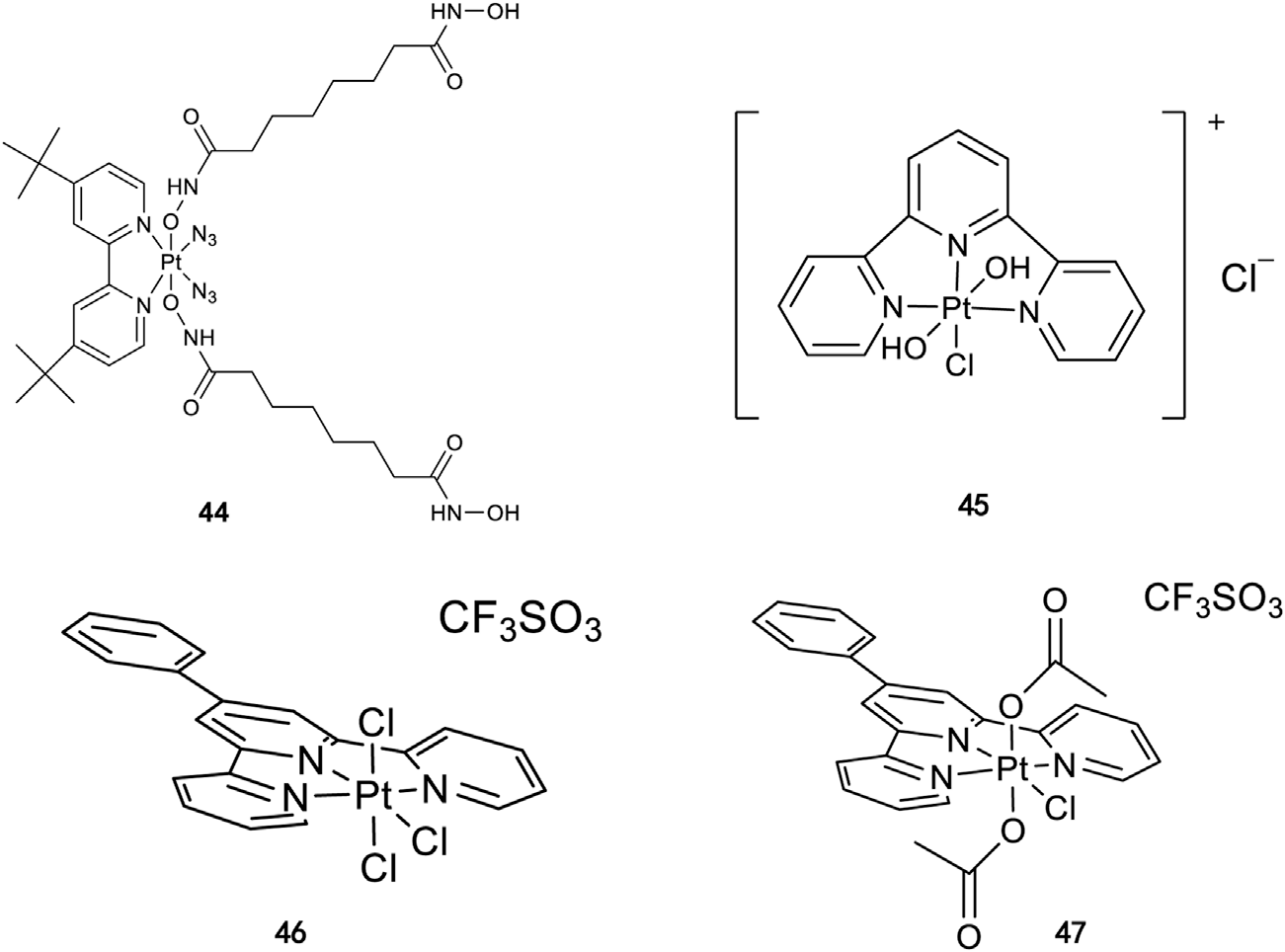
Chemical structure of complexes **44**–**47** [adapted from Kasparkova et al. (2015); Gabano et al. (2017); Canil et al. (2019)].

Similarly, high photoactivated activities over dark activities (PI values >2) were also observed for most of this series of complexes, indicating that the introduction of nitrogen heterocyclic ligands into Pt(IV) prodrugs as non-leaving ligand leads to significant improvement to dark stability of the Pt(IV) prodrugs, compared to the aliphatic amine Pt(IV) prodrugs. It is expected that the introduction of larger heterocyclic ligands, for example, 2,2′-bipyridine (bpy) in *trans,cis*-[Pt(bpy)(OAc)_2_(N_3_)_2_] and 1,10-phenanthroline (phen) in *trans,cis*-[Pt(phen)(OAc)_2_(N_3_)_2_] would reduce the photolysis energy required for Pt(IV) prodrug. Indeed, both of the complexes mentioned before could be activated by a green light at 514 nm (Mackay et al., 2 009b). However, no photocytotoxicity data for them are available. Thus, the bpy and phen complexes are not numbered in this work for further discussion. Kasparkova et al. (2015) designed and synthesized complex **44** based on bipyridine platinum compound (Figure 3), which acts synergistically through two independent mechanisms of action. Under irradiation at 365/420 nm, this complex was activated and acted as both a Pt(II) anticancer drug and histone deacetylase inhibitor, resulting in a better photocytotoxicity against cisplatin-resistant cells (A2780cis) (Nakabayashi et al., 2007).

Following the synthesis of Pt(IV) complexes containing pyridine ligand, terpyridine-based Pt(IV) prodrugs have also been synthesized and reported. For example, Ravera et al. synthesized a terpyridine-based Pt(IV) prodrug, [Pt^(IV)^Cl(OH)_2_(terpy)]^+^ (**45**) (Gabano et al., 2017). This complex is likely a precursor or an intermediate linking to nanoporous silica nanoparticles, of which the IC_50_s against A2780 (7.01 ± 0.56 μM) and sarcomatoid MM98 (10.5 ± 0.1 μM) cell lines are higher than those of cisplatin toward the same cell lines (0.5 ± 0.1 μM and 3.2 ± 1.0 μM, respectively), while its IC_50_ toward cisplatin-resistant MM98R subline (8.09 ± 0.91 μM) is lower than that of cisplatin (19.4 ± 2.8 μM). However, it was less active than its reduced species [Pt^(II)^Cl(terpy)]^+^ and the corresponding silica conjugate (Gabano et al., 2017). Gabbiani et al. also obtained two terpyridine Pt(IV) photocytotoxic anticancer complexes [PtCl_3_(4′-phenyl-2,2′:6′,2″-terpyridine)][CF_3_SO_3_] (**46**) and [Pt(OCOCH_3_)_2_Cl(4′-phenyl-2,2′:6′,2″-terpyridine)][CF_3_SO_3_] (**47**), by using two different oxidation agents, PhICl_2_ and H_2_O_2_, respectively (Canil et al., 2019). These two complexes are physiologically stable with the latter being more stable even in the presence of large excess of GSH. In line with this is the lower reduction potential of **46** (−0.29) compared to **47** (−0.64), which was also thought to be the main reason for the higher IC_50_ of **47** toward A2780 and A2780cis ovarian cell lines in the dark. In contrast, complex **47** showed an excellent photocytotoxicity to the two cancer cell lines (15 ± 1 μM for A2780 and 22 ± 1 μM for A2780cis) under irradiation of 365-nm light.

Nakabayashi et al. (2007) also reported three 2,2′-bipyridine (2,2′-bpy)-based Pt^IV^ complexes, *mer*-[PtCl_3_(2,2′-bpy)(MeNH_2_)]ClH_2_O, *trans*-[PtCl_2_(2,2′-bpy)(MeNH_2_)_2_]Cl_2_, and *trans*-[Pt(2,2′-bpy)(MeNH_2_)_2_(OH)_2_]Cl_2_(MeNH_2_ = methylamine). The former two complexes are not stable in the dark and can undergo hydrolysis of the Cl group, while even under ordinary daylight, photo-induced ligand isomerization occurs with the equatorial methylamine ligand(s) moving into axial positions. Unlike the former two, the latter one was proved to be stable in aqueous solution in the dark and only a trace amount of MeNH_2_ release was observed by NMR within a period of several days at room temperature. However, no cytotoxicity data have been obtained. Therefore, these three complexes are not numbered in this work either.

It is notable that the *trans*-diam(m)ine diazido Pt(IV) complexes, **26**, **28**, and **30**, are more potent than the corresponding *cis*-isomers, **25**, **27**, and **29** (Table 5). For example, the IC_50_ values of *cis*-complex **25** (Farrer et al., 2010a) toward HaCaT, A2780, and A2780cis cells are nearly 15-fold, 42-fold, and 6-fold higher, respectively, than those of the *trans*-complex **26** (Mackay et al., 2007) regardless of irradiation at UV or visible light. This again breaks the paradigm of the structure–activity relationship established on the clinical drug cisplatin and the clinical ineffective transplatin, though the photocytotoxicity of all three *cis*-platinum complexes (**25**, **27**, and **29**) was still much higher than cytotoxicity of cisplatin (13- to 80-fold) (Mackay et al., 2009a; Farrer et al., 2010a).

For the *trans*-complexes, the effect of methyl-substituted pyridine on the activities of Pt(IV) prodrugs was also studied. The complex **30** with meta-methyl-substituted pyridine and complex **31** with para-methyl-substituted pyridine were shown to have higher photocytotoxicity activated at 365 nm than complex **28** with ortho-methyl-substituted pyridine and complex **26** with non-substituted pyridine toward HaCaT and A2780cis cells, though complex **28** has higher dark stability (Table 5) (Farrer et al., 2010a). The substitution of methyl groups in pyridine shows ability to increase the reactivity of diazido Pt(IV) prodrugs to biological targets; however, the ortho substitution may exert negative effects due to its larger steric hindrance. Interestingly, when a relative smaller bromine atom was further modified at the ortho position of the meta-methyl pyridine in complex **32**, the activity of the complex was still lower than that of complexes **30** and **31**. These suggested that the steric effect and the electronic effect on the non-leaving pyridine ligands may jointly influence the activity of diazido Pt(IV) prodrugs (Farrer et al., 2010a).

In addition to the pyridine ring, the heterocyclic penta-ring, thiazole, also brings great improvements in the cytotoxicity of diazido Pt(IV) prodrugs compared to Pt(IV) complexes bearing aliphatic amine ligands. For instance, complexes **33** and **35** exhibited higher photocytotoxicity than complex **26**, especially to A2780cis cells under 365-nm light irradiation (Table 5). Both pyridine and thiazole ligands can sustain a good dark stability, making the photoactivity of these Pt(IV) prodrugs more controllable.

Moreover, in addition to the complexes with monosubstitution of one amine ligand by pyridine or thiazole in diazido Pt(IV) prodrugs, complexes (**36**–**43**) with di-substitution of both amines by dual thiazoles, imidazoles, pyridines, or methyl pyridines were also reported, and their anticancer activities under 420-nm light irradiation were measured (Zhao et al., 2013; Pizarro et al., 2014; Shaili et al., 2019). It was demonstrated that the introduction of dual heterocycle ligands made these target complexes more readily activated by longer wavelength of light, while maintaining high dark stability. The ortho-methyl effect mentioned before still works on these di-pyridine-substituted diazido Pt(IV) prodrugs where complex **37** was the most active prodrug toward both A2780 and A2780cis cells, among complexes **38**–**41**. Of note that di-imidazole ligands in complex **43** showed less efficiency to promote the activity of Pt(IV) prodrugs than thiazole and pyridine ligands. The IC_50_ of complex **43** to A2780 under light irradiation is nearly 10-fold higher than that of other analogs, such as complexes **40–42**, and complex **43** has almost no activity to the A2780cis cell line (Table 5).

The non-substituted am(m)ine non-leaving ligands play significant roles in the action mechanism of Pt(II) anticancer drugs, cisplatin, carboplatin, and oxaliplatin, for example, *via* forming hydrogen bonds with guanine–oxygen to sustain their binding selectivity and strength (Johnstone et al., 2016). While in photoactivatable Pt(IV) prodrugs, similar am(m)ine and aliphatic amine ligands did not satisfy or reach the requirements for the photoactivation of the inert prodrugs, though these am(m)ine ligands could sustain the dark stability of the Pt(IV) complexes. The nitrogen-containing heterocyclic ligands as non-leaving ligands can provide other functions such as increasing the exciting light wavelength, enhancing photoactivities, and promoting PI values of photoactivatable Pt(IV) prodrugs, of which the photolytic products are expected to have distinct action mechanisms from conventional Pt(II) drugs as described before. However, two concerns, difficult synthesis and poor solubility, should be paid much attention for the rational design of the Pt(IV) prodrugs containing large heterocyclic arene ligands. The difficulty of synthesis is mostly laid on the oxidation of the Pt(II) moiety for which harsh conditions and special oxidative agents should be appropriately chosen and applied, while poor solubility resulting from the large heterocyclic arene ligands requires a comprehensive balance between hydrophilicity and hydrophobicity of the non-leaving ligands.

## Axial Ligand

In octahedral Pt(IV) prodrugs, the existence of the axial ligand stabilizes the platinum center and brings more flexibility in the design and synthesis of novel photoactive Pt(IV) prodrugs. In the first generation of iodide Pt(IV) prodrugs [**11**, **12**, **48** (*cis,trans*-{PtCl_2_I_2_(en)}), and **49** (*trans,cis*-{Pt(OSO_2_CH_3_)_2_I_2_(en)})], simple modifications of the axial ligands were achieved (Kratochwil and Bednarski, 1999b), but they did not significantly improve the photoactivity of target complexes. Meanwhile, the as-prepared Pt(IV) complexes had high reduction potential, making them easily reducible, thus leading to poor dark stability (Štarha et al., 2019).

### Cisplatin-Based Pt(IV) Prodrugs

Although cisplatin-bearing dual-chloride leaving ligands are not photoactive, the modification of the axial hydroxyl ligands after their oxidation gives their photochemical potential. Such oxidized species, *cis,cis,trans*-[Pt^IV^(NH_3_)_2_(Cl)_2_(OH)_2_], namely, oxoplatin, is the starting structure of the popular cisplatin-based Pt(IV) prodrugs.

**FIGURE 4:**
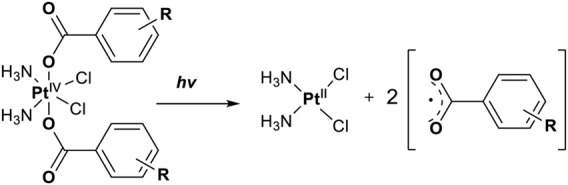
Schematic representation of photolysis of complexes **50**, **56**, and **57** [adapted from Lee et al. (2019)].

In 2014, Chee Fei Chin in PhD thesis reported six Pt(IV) prodrugs (**50–55)** containing ester bonds generated by the interaction between hydroxyl and carboxylic acid and explored the influence of the axial ligands on the maximum absorption wavelength of the resulting complexes (Table 6) (Fei, 2014). It was demonstrated that the introduction of the benzene ring resulted in an obvious increase in the maximum absorption wavelength (from 204 to 226 nm), while the introduction of pyrene made the maximum absorption wavelength more significant red-shift (from 204 to 344 nm). However, the similar maximum absorption wavelength of complex **55** (*λ* = 345 nm) and complex **53** (*λ* = 345 nm) indicates that the increasing conjugation from the aromatic ligands was not the mere factor determining the red-shift interval. Lee et al. (2019) designed and synthesized complexes **56** and **57**. Compared to **51**, complexes **50**, **56**, and **57** have axial aryl groups (Table 6), which increases the ultraviolet absorption and stabilizes the dissociated carboxyl units, thus favoring the photoactivated reduction of platinum (Figure 4). During the photoactivation, complex **50** produced complicated ESI-MS spectra due to the lack of aromatic axial ligands, while complex **57** showed a faster photoreduction rate than complex **51**. Benzoyl radical, which was the first evidence of carboxyl radical species formed *via* photoactivation of non-azido Pt(IV) complexes, was trapped by 5,5,5-dimethyl-1-pyrroline N-oxide (DMPO) during photoactivation of **50**. However, it seemed useless to the anticancer properties as it was quickly quenched by a solvent.

**TABLE 6:** Chemical structures of complexes **47**–**56**.

	Complex	R1	R2
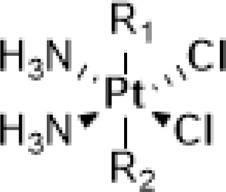	**50**	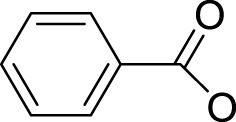	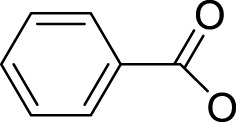
	**51**	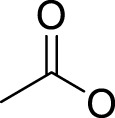	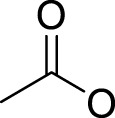
	**52**	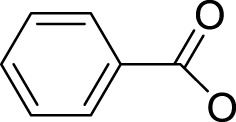	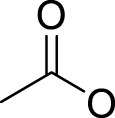
	**53**	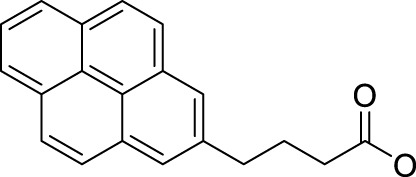	OH
	**54**	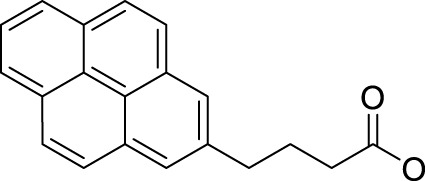	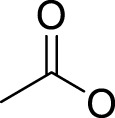
	**55**	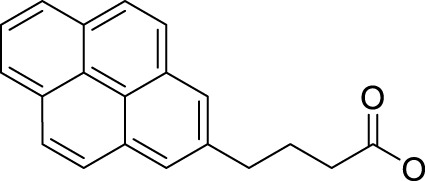	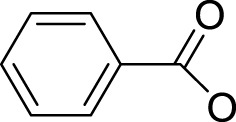
	**56**	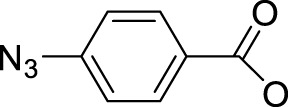	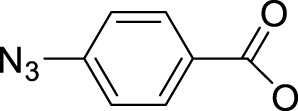
	**57**	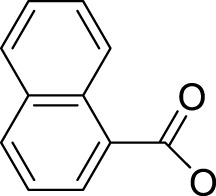	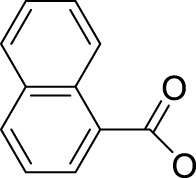
	**58**	OH	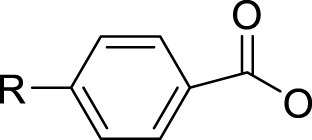
	**59**	OH	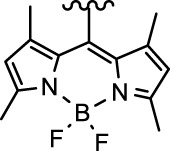

The bold values represent the numbering of the complexes.

Bera et al. (2021) prepared the complex oxoplatin-B by axial derivatization with a fluorescent BODIPY ligand (**58**) (Table 6). A similar structure but with a methyl modification at the axial benzoic acid ligand was also synthesized as a control (**59**). The BODIPY moiety of complex **58** with medium quantum yield can be used for cell imaging, particularly mitochondrial localization, which may improve the selectivity of this complex as a prodrug. Complex **58** showed an absorption band at 500 nm and an emission band at 515 nm in 1% dimethyl sulfoxide/Dulbecco’s modified Eagle’s medium (pH = 7.2). Upon visible light irradiation (*λ* = 400–700 nm, 10 J cm^−2^), complex **58** generated singlet oxygen and showed more cytotoxicity toward human breast (MCF7), cervical (HeLa), and lung (A549) cancer cells than complex **59**, BODIPY ligand alone, cisplatin, and itself in dark. Singlet oxygen generation is one of the objectives of the authors to conjugate a BODIPY to the Pt(IV) center. The ROS generated by **58** showed promising photo-induced cleavage efficiency on supercoiled (SC) plasmid pUC19 DNA (2,686 base pair) with 1 h irradiation, leading to formation of ∼63% nicked circular (NC) DNA, whereas no NC DNA formation was observed for complex **59**.

### Carboplatin- and Oxaliplatin-Based Pt(IV) Prodrugs

**FIGURE 5:**
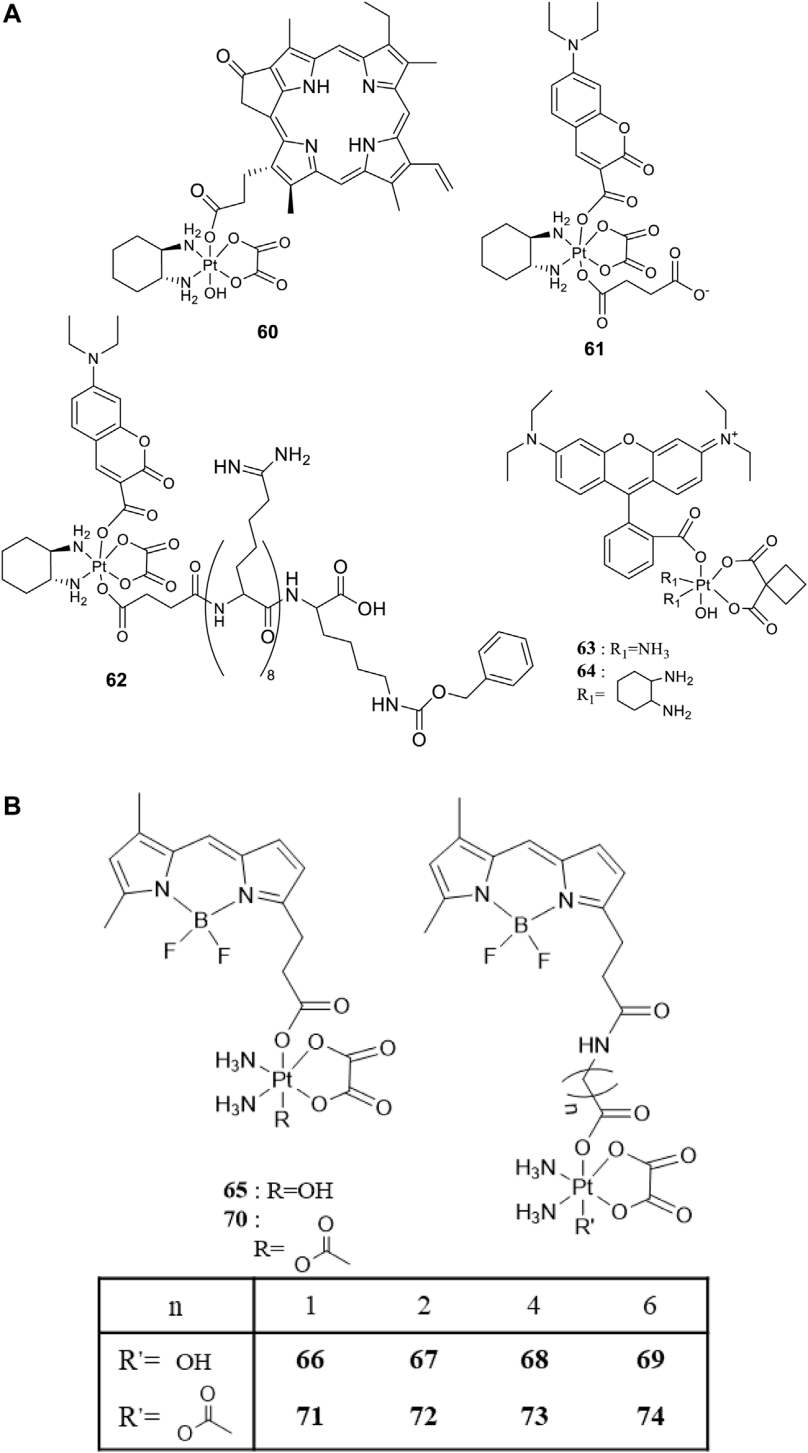
**(A)** Chemical structures of complexes **60–64** [adapted from Wang et al. (2019); Deng et al. (2020); Deng et al. (2021)]. **(B)** Chemical structures of BODIPY-conjugated Pt(IV) prodrugs [adapted from Yao et al. (2020); Yao et al. (2021)].

**FIGURE 6:**
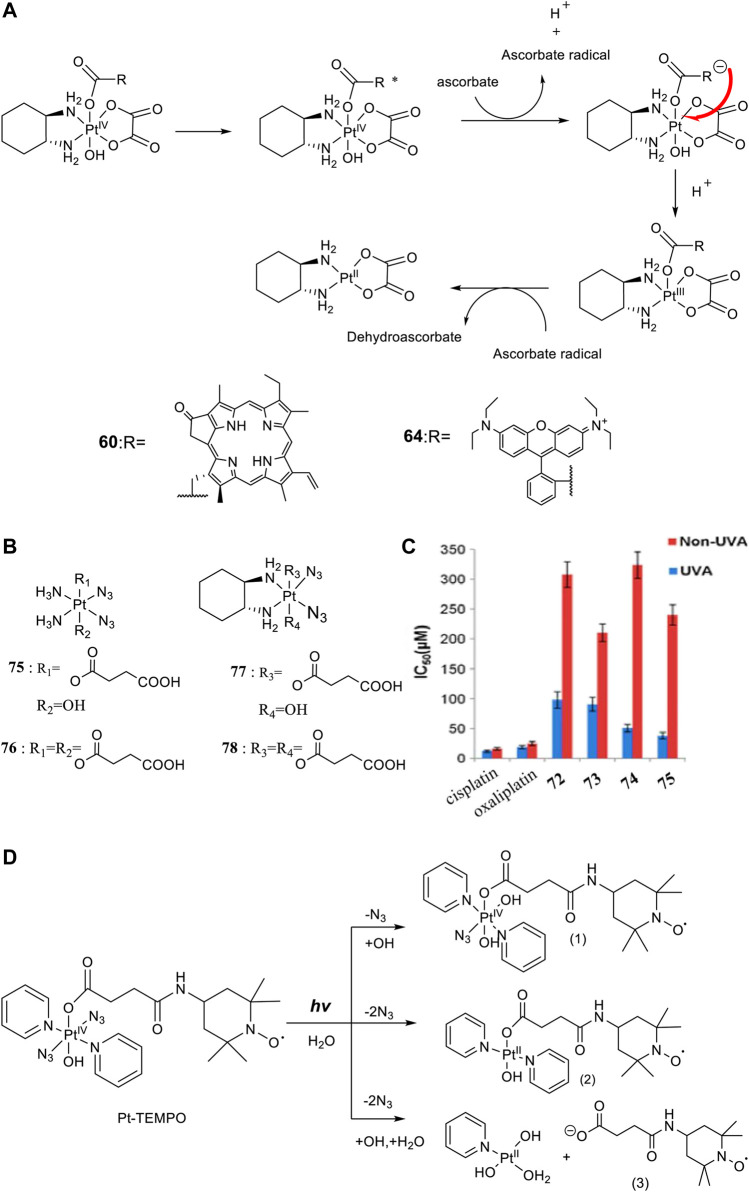
**(A)** Proposed photoreduction mechanism of complexes **60** and **64** in the presence of ascorbate under physiological conditions (adapted from Wang et al. (2019); Deng et al. (2021)). **(B)** Chemical structures of complexes **75–78**. **(C)** IC_50_ values of complexes **75**–**78** against SKOV-3 cells as determined by MTT assay [reproduced from Xiao et al. (2014)]. **(D)** Photoreaction pathways of Pt-TEMPO (0.1 mM) in H_2_O upon irradiation of blue visible light [adapted from Venkatesh et al. (2016)].

For developing photoactivatable carboplatin- and oxaliplatin-based Pt(IV) prodrugs, Guangyu Zhu′s team made a lot of efforts and obtained a series of multifunctional promising Pt(IV) prodrugs. In 2019, they designed and synthesized an oxaliplatin-based Pt(IV) prodrug [Pt^IV^(DACH)(PPA)(OH) (ox)] (**60**; DACH = (1R,2R)-1,2-diaminocyclohexane, ox = oxalate), namely, phorbiplatin (Wang et al., 2019). In complex **60**, the pyropheophorbide a (PPA) ligand occupied one of the axial positions with hydroxyl at another (Figure 5A), allowing the Pt(IV) complex activatable by a red light at 660 nm. The axial PPA ligand functions as both a photosensitizer and a photocatalyst with long absorption wavelength (650 nm) and good electron transfer ability. However, PPA could not reduce the Pt(IV) center in phorbiplatin directly. Instead, PPA itself was reduced first under irradiation and in the presence of biological reductants, such as ascorbate, 2-(N-morpholino) ethanesulfonic acid (MES), GSH, or DNA. The reduced PPA in turn reduced the Pt(IV) center to complete the photoreduction of the Pt(IV) prodrug (Figure 6A). Phorbiplatin is stable and shows little cytotoxicity in the dark, while upon irradiation with red light the complex exhibits promising anticancer activities both *in vitro* and *in vivo*. The photocytotoxicity of phorbiplatin was nearly 1786-fold higher than that of oxaliplatin upon irradiation (Table 7). Such high photocytotoxicity of phorbiplatin was thought to be the combination of three factors, that is, high accumulation of Pt in cancer cells, generation of active Pt(II) species, and generation of ROS including singlet oxygen and hydroxyl radicals under irradiation. Complex **60** was thought to be the first small-molecule Pt(IV) prodrug that can be efficiently and controllably activated by red light, and no damage to the liver and other organs when treated with this complex under irradiation, implicating its huge potential in clinic.

**TABLE 7:** IC_50_ values of complexes **60—74** to various cancer cell lines.

Complex	λ_max_/nm of the LMCT band (N_3_→Pt)	λ_irr_/nm	Cell line	IC_50_ (light)/μM	IC_50_ (dark)/μM	PI IC_50_ (dark)/IC_50_ (light)	Reference
60	400–450	650	A2780	0.13 ± 0.01	>10	76.9	Wang et al. (2019)
			A2780cisR	0.19 ± 0.01	>10	52.6	
			MCF-7	0.044 ± 0.004	>10	227.3	
			4T1	0.13 ± 0.004	>10	76.9	
			MRC-5	—	>10	—	
61	400–500	450	HCT116 (p53+/+)	52.8 ± 6.6	>100	1.9	Deng et al. (2020)
			HCT116 p53−/−	89.3 ± 5.6	>100	1.1	
			HT29	81.5 ± 5.5	>100	1.2	
			HeLa	61.8 ± 4.8	>100	1.6	
			MCF-7	86.2 ± 9.3	>100	1.2	
			MDA-MB-231	79.4 ± 6.6	>100	1.3	
			A2780	56.2 ± 5.6	>100	1.8	
			A2780cisR	>100	>100	—	
			A549	81.3 ± 6.7	>100	1.2	
			A549cisR	>100	>100	—	
			MRC5	—	>100	—	
62	400–500	450	HCT116 (p53+/+)	0.9 ± 0.2	47.1 ± 5.9	52	Deng et al. (2020)
			HCT116 p53−/−	1.3 ± 0.3	80.6 ± 7.1	62	
			HT29	2.7 ± 0.4	51.6 ± 4.2	19	
			HeLa	5.6 ± 1.1	38.8 ± 4.2	7	
			MCF-7	5.5 ± 2.1	52.1 ± 6.9	10	
			MDA-MB-231	8.1 ± 3.0	69.3 ± 5.2	9	
			A2780	3.9 ± 0.6	42.1 ± 4.7	11	
			A2780cisR	4.9 ± 0.8	72.6 ± 4.8	15	
			A549	6.9 ± 1.1	49.2 ± 4.2	7	
			A549cisR	4.0 ± 0.5	114.8 ± 7.7	29	
			MRC5	—	93.9 ± 6.8	—	
63	500–600	Visible light (400–760 nm)	A2780	44 ± 5	220 ± 17	5.0	Deng et al. (2020)
			A2780cisR	41 ± 5	250 ± 18	6.1	
			MCF-7	77 ± 9	245 ± 17	3.2	
			A549	57 ± 5	251 ± 15	4.4	
			A549cisR	61 ± 6	289 ± 17	4.7	
			HCT116	47 ± 5	248 ± 18	5.3	
			MRC-5	—	>300	—	
64	500–600	Visible light (400–760 nm)	A2780	25 ± 2	108 ± 9	4.3	Deng et al. (2021)
			A2780cisR	20 ± 7	136 ± 13	6.8	
			MCF-7	43 ± 3	133 ± 11	3.1	
			A549	29 ± 5	104 ± 15	3.6	
			A549cisR	33 ± 5	142 ± 10	4.3	
			HCT116	18 ± 1	112 ± 6	6.2	
			MRC-5	—	116 ± 7	—	
65	504	White light	A2780	>100	>100	—	Yao et al. (2020)
66	503	White light	A2780	43.9 ± 5.2	69.8 ± 4.9	1.6	
67	503	White light	A2780	48.3 ± 2.0	74.8 ± 8.2	1.5	
68	503	White light	A2780	76.8 ± 9.9	>100	1.3	
69	504	White light	A2780	>100	>100	—	
70	505	White light	A2780	66.6 ± 7.0	>100	1.5	Bera et al., (2021); Wang et al., (2019)
		Green light	MCF-7	173.4 ± 8.8	15.7 ± 1.0	11.0	
			MDA-MB-231	162 ± 8.4	19.1 ± 4.7	8.5	
			SKOV3	>200	22.4 ± 0.8	>8.9	
			A2780	68.5 ± 5.0	31.0 ± 7.1	2.2	
			HeLa	>200	37 ± 1.5	>5.4	
			A549	148.9 ± 17.3	60.5 ± 14	2.5	
			WI-38	>200	—	—	
71	503	White light	A2780	43.8 ± 3.9	>100	2.3	Wang et al. (2019)
72	503	White light	A2780	88.6 ± 15.7	>100	1.1	
73	504	White light	A2780	>100	>100	—	
74	504	White light	A2780	>100	>100	—	

To enable the prodrugs more photo-controllable, in 2020, Zhu and co-workers further designed and synthesized an oxaliplatin-based photocaged Pt(IV) prodrug, complex **61**, by introducing a coumarin derivative at the axial position, as shown in Figure 5A (Deng et al., 2020). The wavelength (450 nm) activating complex **61** is shorter than the red light used to activate complex **60**, and the photocytotoxicity of **61** is only two orders of magnitude higher than that of oxaliplatin. However, complex **61** is very stable in the dark under physiological conditions even in the presence of 2 mM sodium ascorbate, where 93% of **61** remained intact after 24-h incubation. Unexpectedly, water oxidation was involved in the light activation process of this complex accompanied with oxygen generation but without radical formation. Such photoactivation pathway *via* water oxidation was rare. The proposed mechanism was that first the Pt-O bond of axial ligand in complex **61** was cleaved to produce the free axial ligands and cationic Pt(IV) intermediate upon irradiation; second, two electrons transferred from water to reducing Pt(IV) accompanied with formation of oxaliplatin, two protons and oxygen. Furthermore, Zhu et al. attached a cell-penetrating peptide, R_8_K, at another axial position through a succinic acid linker to develop the so-called coumaplatin (complex **62**, Figure 5A). Complex **62** accumulated very efficiently in the nucleoli and induced cell senescence and immunogenic cell death along with T-cell activation through the entire coumaplatin instead of the reduced oxaliplatin species. Such photo-caged, water-oxidizing, and nucleolus-targeted trifunctional Pt(IV) anticancer prodrug represents a very effective and attractive strategy for designing and developing Pt(IV) prodrugs.

Later, rhodamine B (RhB), a widely used fluorescent dye, was employed as one of the axial ligands to synthesize photoactivatable Pt(IV) prodrugs **63** and **64**, named rhodaplatin 1 (carboplatin-based structure) and rhodaplatin 2 (oxaliplatin-based structure), respectively (Figure 5A) (Deng et al., 2021). The RhB ligand serves as an intramolecular photoswitch ligand, showing many advantages over the conventional “photocatalyst plus Pt(IV) substrate” combination, for example, enabling the complexes more efficient accumulation and significant colocalization in cancer cell. The conjugation of RhB as an axial ligand promoted the photoconversion efficiency of the prodrugs by 4.8 × 10^4^-fold, contributable to increased proximity to the Pt(IV) center through the covalent bond. This leads to increased photocytotoxicity of the complexes to cancer cells including resistant cancer cells to conventional Pt(II) drugs. The activation of rhodaplatin 2 (**64**) includes two important electron transfer steps: the first electron transfer from ascorbate to RhB ligand to form the Pt(IV) intermediate containing the RhB radical(III), and the second electron transfer from RhB ligand to Pt(IV) center to produce Pt(III) intermediate along with the release of a hydroxyl group (Figure 6A). Moreover, complex **64** showed an efficient and preferred accumulation in the mitochondria, inducing the mitochondria DNA damages and showing higher anticancer activity than the corresponding carboplatin-based rhodaplatin 1 (**63**). Nevertheless, the anticancer activity of carboplatin-based Pt(IV) prodrug **60** is still higher than carboplatin and oxaliplatin. This inspired the research group to design and synthesize another types of carboplatin-based Pt(IV) prodrugs (**65–74**, Figure 5B) using BODIPY derivatives as axial leaving ligands (Yao et al., 2020). These complexes were divided into two subgroups differing in that one of the axial ligands was a hydroxyl (**65–69**) or acetyl (**70–74**) group, and another axial position was occupied by BODIPY derivatives *via* a carbon-chain linker in different length. All the as-prepared complexes have a LMCT band (N_3_→Pt) at around 503 or 504 nm and can be activated by white light, showing a moderate photoactivated anticancer activity to A2780 cells with the lowest IC_50_ as 43.9 ± 5.2 μM for complex **66** and 43.8 ± 3.9 μM for complex **71**. This group of carboplatin-based prodrugs were less photocytotoxic than oxaliplatin-based analogs and even much less active than that of cisplatin-based BODIPY Pt(IV) prodrug (**60**). Except complexes **65** and **69**, a linker- length-dependent activity was observed in which increased carbon-chain length negatively regulated the anticancer activities for both the subgroups of complexes upon light irradiation. However, the variation of hydroxyl and acetyl axial ligands had little effect on the photocytotoxicity of the whole group of carboplatin-based complexes (Table 7) (Yao et al., 2021).

### Diazido Pt(IV) Prodrug Derivatives

The emergence of diazido Pt(IV) anticancer prodrugs have attracted great interests due to their high dark stability and promising photoactivated activity. This spurs broader investigations on the designs and developments of novel diazido Pt(IV) prodrugs mostly through the axial modifications. One of the simplest derivation ways is attaching one or two succinic anhydrides to the axis positions based on the *cis*-NH_3_ and *cis*-DACH-type of diazido Pt(II) complexes (DACH = 1,2-cyclohexanediamine). Although this series of diazido Pt(IV) prodrugs, that is, complexes **75**–**78** (Figure 6B), were thought to be intermediates, they showed high dark stability (Figure 6C), evidenced by constant presence of the N_3_→Pt transition absorption band at 258 nm in aqueous solution in the dark for 90 days (Xiao et al., 2014), while they can be activated under 365-nm UVA irradiation to release one or two succinic acid moieties. Moreover, complexes **77** and **78** bearing DACH ligands were more readily activated by UVA irradiation than complexes **75** and **76** bearing NH_3_ ligands, indicated by the longer T_50_ values (time for 50% degradation of Pt(IV)-N_3_) for the latter two. Unfortunately, despite of a nearly equal cellular uptake to and much lower dark cytotoxicity of **75**–**78** than cisplatin and oxaliplatin, the photocytotoxicity upon UVA irradiation of **75**–**78** are much lower than the cytotoxicity of cisplatin and oxaliplatin to SKOV-3 cells. Importantly, the Pt(IV) prodrugs derived from the oxaliplatin structure were found to be about 2- to 5-fold more photocytotoxic than the corresponding cisplatin-based prodrugs (complexes **77** and **78** versus complexes **75** and **76**).

Actually, apart from cisplatin- or oxaliplatin-derived diazido types of Pt(IV) complexes described before, most of the modifications on the diazido Pt(IV) prodrugs were based on the type of [Pt(N_3_)_2_(OR_1_)(OR_2_)(Py)_2_] complexes with two pyridines as the non-leaving ligands. This makes almost all this type of Pt(IV) prodrugs activatable by blue light or green light. Meanwhile, as the *trans*-Pt(IV) complexes are usually more cytotoxic than the corresponding *cis*-complexes (Farrer et al., 2010a), most of the derivation focused on the basis of *trans*-diazido Pt(IV) prodrugs. For example, the TEMPO (2,2,6,6-tetramethylpiperidine-1-oxyl), a spin-labeling nitroxide, was successfully conjugated to the axial hydroxyl through a succinate linker to produce the first example of diazido Pt(IV) prodrug, *trans,trans,trans*-[Pt(N_3_)_2_(OH)(OCOCH_2_CH_2_CONH-TEMPO)(Py)_2_], namely, Pt-TEMPO (Figure 6D) with ability to release nitroxide/azidyl radicals as well as Pt(II) species upon irradiation of blue visible light at 465 nm (Venkatesh et al., 2016). The introduction of the TEMPO moiety even extended the photoactivation wavelength to green light at 517 nm. UV-Vis spectroscopy monitoring the LMCT band absorption under 420-nm irradiation observed only one photodecomposition pathway in which there was a loss of an azido ligand and/or electron transfer from azido ligands to Pt(IV), resulting in the formation of azidyl radicals and Pt(IV/II) species. However, ESI-MS identified photolytic products generated by three pathways. This is an obvious clue that a substrate-dependent photodecomposition mechanism may exist for Pt-TEMPO prodrugs, similar to that for complex **36**, nevertheless which needs further exploration. The photoactivity of Pt-TEMPO to A2780 human ovarian carcinoma cells is as high as the organic photo-sensitizer chlorpromazine (CPZ), which may result from attacking DNA by the toxic Pt(II) photoproducts, combining with the activity of the reactive azidyl and TEMPO radicals.

**FIGURE 7:**
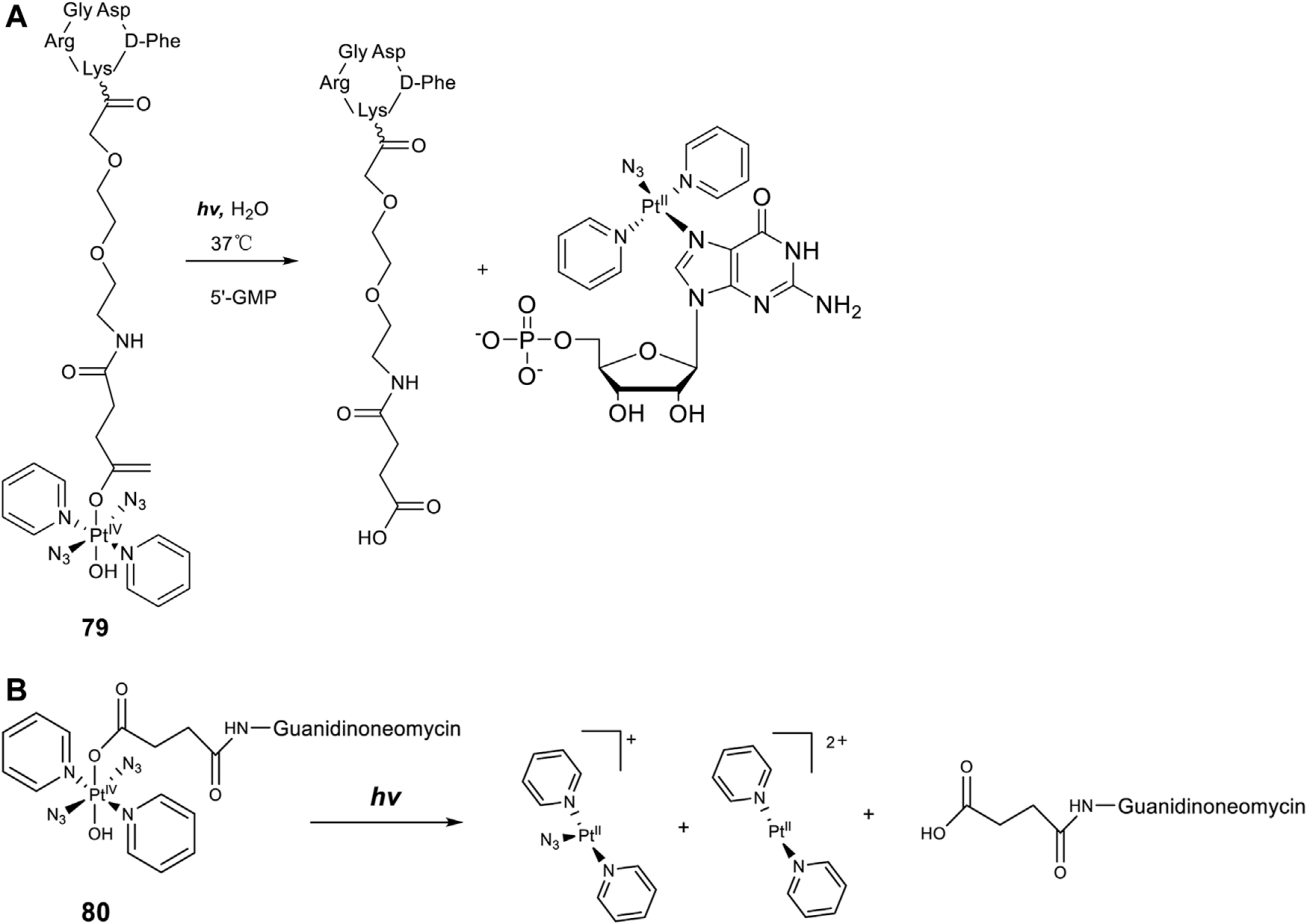
**(A)** Schematic diagram of the photoreaction of complex **79** with 5′-GMP [adapted from Shaili et al. (2015)]. **(B)** Schematic representation of the photodissociation process of complex **80** [adapted from Shaili et al. (2015)].

Axial modifications can introduce multifunctions to diazido Pt(IV) prodrugs, especially with specific targeting moieties. Gandioso et al. (2015) synthesized a new Pt(IV) prodrug (**79**, Figure 7A) by linking a cyclic RGD (Arg-Gly-Asp) peptide to [Pt(N_3_)_2_(OH)_2_(Py)_2_]. The RGD peptide can be selectively recognized by α_V_β3 and α_V_β5 integrins, which are transmembrane heterodimeric glycoproteins overexpress in different tumor cells and involve in tumor angiogenesis (Friedlander et al., 1995; Desgrosellier and Cheresh, 2010). These receptor-targeting Pt(IV)-based anticancer drugs have a dually controlled selectivity and showed higher intracellular accumulation and phototoxicity to SK-MEL-28 melanoma cancer cells, which overexpress α_V_β3 and α_V_β5 integrins, than to DU-145 human prostate carcinoma cells upon blue light irradiation. One major product, *trans*-[Pt(N_3_)(5′-GMP)(py)_2_]^+^, and two minor products, *trans*-[Pt(py)_2_(5′-GMP)_2_]^2+^ and [Pt_2_(N_3_)(py)_4_(5′-GMP)]^+^, were identified by ESI-MS during the photoactivation (*λ*_irr_ = 420 nm, 11 mW cm^−2^, 45 min, 37°C) of **79** in the presence of 2 mol equiv. 5′-GMP. More importantly, the released intact succinate-peptide did not compete with 5′-GMP for binding to Pt(II) species but serving as a simple targeting vector of the Pt(IV) prodrug. Such an integrin-targeting approach provides a new strategy to improve the selectivity and reduce the toxicity of Pt(IV) prodrugs, spurring other similar beneficial developments.

In a similar approach to the receptor-targeting rationale, a RNA-targeting photosensitive diazido Pt(IV) prodrug (**80**, Figure 7B) was designed and synthesized by the same research group by conjugating guanidinoneomycin, a well-known RNA binding ligand, at the axial position through a succinate linker (Shaili et al., 2015). Apart from the released platinum(II)-based cytotoxic species upon visible-light irradiation, the authors hoped that the guanidinoneomycin vector can potentially promote platination of RNA over DNA, offering a novel mechanism of action for anticancer metallodrugs. High reactivity with 5′-guanosine monophosphate and a 7-mer oligodeoxynucleotide, 5′-dCATGGCT, were probed by mass spectrometry coupled to HPLC. The results showed that the covalent attachment of guanidinoneomycin does not modify the photoactivation properties of **80**, and the guanidinoneomycin potentially help promote the platination of RNA over DNA. Guanidinoneomycin conjugation was showed to indeed improve the intracellular accumulation of complex **81** particularly in SK-MEL-28 cells. Notably in this study, the empirical knowledge that Pt(IV) prodrugs are generally inert to ligand substitution was disturbed, evidenced by the substitutions of one azide and/or another non-modified hydroxyl by trifluoroacetate. However, the trifluoroacetate-substituted complexes, especially the adduct of which both axial ligands were replaced with trifluoroacetate were insoluble in aqueous media.

Recently, Sadler and co-workers have also achieved great progresses in the development of novel diazido Pt(IV) prodrugs. In 2020, they designed and synthesized two diazido Pt(IV) prodrugs (**81** and **82**, Table 8) with biotin as one of the axial ligands and hydroxyl or dichloroacetate (DCA), a pyruvate dehydrogenase kinase (PDK) inhibitor, as another axial ligand (Table 8) (Shi et al., 2020b). Both the complexes exhibited excellent dark stability. Upon 1-h light irradiation at 465 nm, complex **82** bearing biotin and DCA as axial ligands showed 5-fold higher photocytotoxicity than its precursor *trans,trans,trans*-[Pt(N_3_)_2_(OH)_2_(py)_2_] (**36**) with two hydroxyl groups as axial ligands, and at least 2-fold higher photocytotoxicity than complex **81** with biotin and hydroxyl as axial ligands to A2780 cell lines, contributable to the synergistic anticancer activity of the released DCA ligand. The conjugation of biotin alone was not helpful to the cellular uptake of complex **81** in A2780 cells, while the substitution of the second axial hydroxyl ligand with DCA significantly increase Pt cellular uptake of the resulting complex **82**, probably due to higher lipophilicity of **82** (Shi et al., 2020b) over the mono-substituted complex **81** (IC_50_: 11.7–21.1 μM) (Table 8).

**TABLE 8:** Chemical structures of complexes **81–94** and IC_50_ values of complexes toward different cell lines.

	Complex	R_1_	R_2_	λ_max_/nm of the LMCT band (N_3_→Pt)	λ_irr_/nm	Cell line	IC_50_ (light)/μM	IC_50_ (dark)/μM	PI IC_50_ (dark)/IC_50_ (light)	Reference
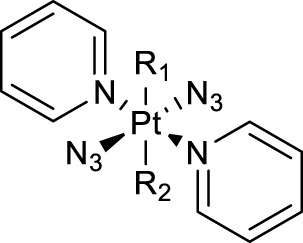	**81**	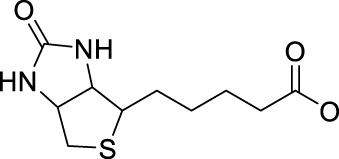	OH	260	465	A2780	11.7 ± 0.3	>100	>8.5	Shi et al. (2020b)
						A549	13.3 ± 0.7	>100	>7.5	
						PC3	21.1 ± 0.4	>100	>4.7	
						MRC5	—	>100	—	
	**82**	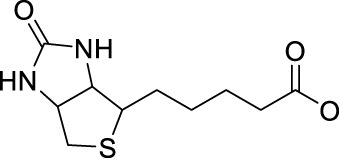	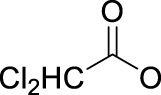	308	465	A2780	1.3 ± 0.2	>50	>38.5	—
						A549	5.9 ± 0.6	>100	>16.9	
						PC3	3.0 ± 0.1	>100	>33.3	
						MRC5	—	>100	—	
	**83**	OH	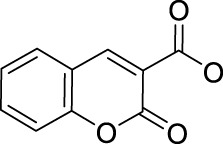	298	465	A2780	2.9 ± 0.2	>100	>34	Shi et al. (2020a)
						A549	7.8 ± 0.1	>100	>12	
						MRC5	—	>100	—	
	**84**	OH	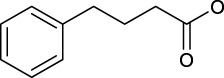	297	465	A2780	0.92 ± 0.07	>100	>108	—
						A549	5.44 ± 0.05	>100	>18	
						MRC5	—	>100	—	
	**85**	OH	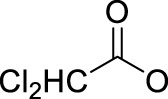	299	465	A2780	1.2 ± 0.2	>100	>83	—
						A549	6.6 ± 1.1	>100	>15	
						MRC5	—	>100	—	
	**86**	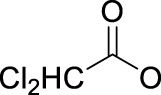	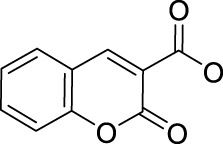	306	465	A2780	0.11 ± 0.02	1.9 ± 0.1	17.3	
						A549	2.6 ± 0.3	>50	>19	
						MRC5	—	>50	—	
	**87**	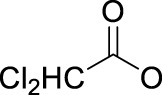	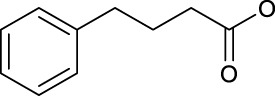	313	465	A2780	0.15 ± 0.01	1.3 ± 0.2	8.7	
						A549	1.2 ± 0.1	>20	>16	
						MRC5	—	>20	—	
	**88**	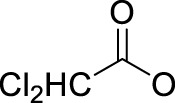	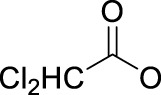	319	465	A2780	0.39 ± 0.01	1.9 ± 0.3	4.9	
						A549	1.9 ± 0.1	>20	>10	
						MRC5	—	>20	—	
	**89**	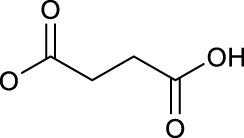	OH	Around 300	420	A2780	2.7	>132.3	>49	Shaili et al. (2021)
						A2780cis	5.4	>199.8	>37	
						OE19	8.2	>172.2	>21	
						SK-MEL-28	15.5	175.2	11.3	
						DU-145	20.0	176.0	8.8	
	**90**	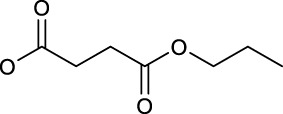	OH	Around 300	420	A2780	3.7	>162.8	>44	Shaili et al. (2021)
						A2780cis	4.0	>164.0	>41	
						OE19	10.3	>154.5	>15	
	**91**	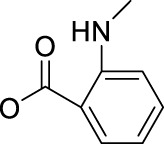	OH	Around 300	420	A2780	13.4	>26.8	>2
						A2780cis	13.0	>26.0	>2
						OE19	17.5	>33.3	>1.9
	**92**	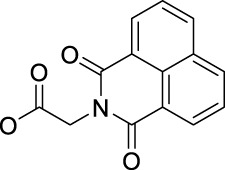	OH	306	—	—	—	—	—	—
	**93**	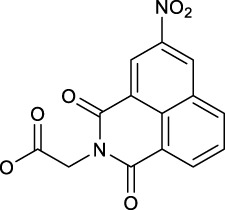	OH	279	465	A549	11.8 ± 0.1	>50	>4.2	—
						PC3	6.4 ± 0.7	>50	>7.5	
	**94**	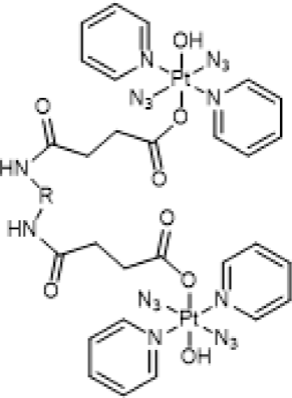	OH	296	465	A549	13.5 ± 1.3	>100	>7.4	—
						PC3	10.1 ± 0.2	>100	>9.9	
						A2780	1.2 ± 0.1	>100	>83.3	
					520	A549	92.8 ± 13.5	>100	>1.1	
						PC3	32.7 ± 0.5	>100	>3.1	
						A2780	35.3 ± 2.2	>100	>2.8	
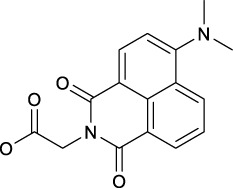	**95**	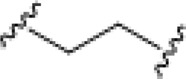	—	Around 300	465	A2780	77.0 ± 2.9	>100	>1.3	Shi et al. (2018)
						A2780cis	29.5 ± 11.0	>100	>3.4	
						OE19	36.2 ± 2.4	41.3 ± 3.2	1.1	
						MRC5	>100	>100	—	
	**96**	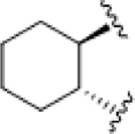	—	Around 300	465	A2780	33.9 ± 5.2	>100	>2.9	—
				Around 300	465	A2780cis	67.6 ± 15.7	>100	>1.5	
				Around 300	465	OE19	27.5 ± 5.1	49.8 ± 1.5	1.8	
				Around 300	465	MRC5	>100	>100	—	
	**97**	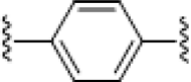	—	Around 300	465	A2780	78.3 ± 6.8	>100	>1.3	—
				Around 300	465	A2780cis	35.5 ± 3.4	>100	>2.8	
				Around 300	465	OE19	43.71.9	53.4 ± 0.4	1.2	
				Around 300	465	MRC5	>100	>100	—	
	**98**	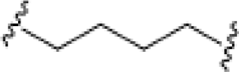	—	Around 300	465	A2780	16.73.3	>100	>6.0	—
				A2780cis	17.0 ± 2.3	>100	>5.8			
				OE19	8.8 ± 0.9	97.0 ± 5.7	11.0			
				MRC5	>100	>100	—			

The bold values represent the numbering of the complexes.

To further investigate the effects of axial functionalization, three mono-functionalized diazido Pt(IV) prodrugs attaching to anticancer agent coumarin-3 carboxylate (cou), PDK inhibitor 4-phenylbutyrate (PhB), or DCA (complexes **83**–**85**, Table 8) and three biaxially functionalized prodrugs with further modification with a DCA ligand (complexes **86**–**88**, Table 8) were developed (Shi et al., 2020a). Notably, mono-functionalized complexes (**83**–**85**) with one hydroxide ligand showed higher aqueous solubility and more negative reduction potential, while significantly higher cellular Pt accumulation (ca. 3–18×) and photo-induced cellular ROS levels were observed for di-functionalized complexes **86**–**88**. However, the axial substituents did not affect the interaction between the platinum center and 5′-GMP upon irradiation**.** Both mono- and di-functionalized complexes displayed higher photocytotoxicity than the parent complex **36** to both A2780 and A549 cancer cell lines, and the di-functionalized complexes were a little more cytotoxic upon the blue light irradiation at 465 nm (Table 8). These again confirmed the synergistic effect of the DCA ligand which were also evidenced by the significantly increased platinum accumulation and photo-generated ROS levels of the di-functionalized complexes over mono-functionalized analogs. However, the much lower aqueous solubility of di-functionalized complexes **86**–**88** should be noted.

Another work from Sadler’s group aimed at exploring the effect of axial ligand modification on the photochemistry and photobiology of diazido Pt(IV) prodrugs. Furthermore, three newly synthesized complexes, *trans,trans,trans*-[Pt(N_3_)_2_(OH)(succ)(py)_2_](succ = succinate; **89**), *trans,trans,trans*-[Pt(N_3_)_2_(OH)(succ-Pr)(py)_2_](succ-Pr = 4-oxo-4-propoxybutanoate; **90**), and *trans, trans,trans*-[Pt(N_3_)_2_(OH)(N-MI)(py)_2_](N-MI = N-N-methylisatoate; **91**) were studied in this work (Shaili et al., 2021). The introduction of N-MI ligand was anticipated to increase the photosensitivity to irradiation with visible light as it has an additional absorption band (ca. 350 nm). It was showed that the disappearing rate of the LMCT (N_3_→Pt) band, which indicates the dissociation of azido ligand(s) upon 517 nm light irradiation, decreased in an order of complex **90** > complex **91** > complex **89** ≫ complex **36**. All the three complexes (**89–91**) exhibited minimal toxicity in the dark, and upon blue light irradiation complexes **89** and **90** exhibited nearly 2-fold higher phototoxicity than complex **36** to A2780 and A2780cis. However, complex **91** was the least phototoxic with an IC_50_ of 13.4 μM, though its ca. 10-fold higher cellular uptake in comparison with other complexes (Table 8). The incorporation of aromatic ligands in the axial position in complex **91** may enhance its passive transport across cell membrane, increasing cellular uptake of **91**, and the low yield of azidyl radicals was thought to be one of the main reasons for its low phototoxicity. The higher cytotoxicity of this kind of complexes were attributed to the combination of versatile action modes, for example, intercalative DNA binding, concentration-dependent interstrand DNA crosslinking, significant cellular accumulation of Pt, and photo-oxidation of biomolecules.

Following this finding that incorporating aromatic ligands could promote the cellular uptake of diazido Pt(IV) prodrugs, Sadler and co-workers attached the fluorescent 1,8-naphthalimide or its NO_2_ and NMe_2_ derivatives at the one of axial positions in complex **36** to produce complexes **92–94** (Table 8) (Shi et al., 2021). All the three complexes can undergo photo-decomposition upon 420 and 463 nm irradiation. Although complex **92** has the highest ability to intercalate into DNA in solution among other two analogs having larger steric hindrance of axial ligands, its aqueous solubility was so poor that photocytotoxicity assay and cellular uptake determination could not be performed on it. This warned us that balancing the hydrophobicity and hydrophilicity when attaching aromatic ligands to the axis of Pt(IV) prodrugs must be considered. The modification on the naphthalimide ligand by the NO_2_ or NMe_2_ group remarkedly improved the aqueous solubility of complexes **93** and **94**, of which the cellular uptake was 15 × higher than that of unmodified complex **36** in A2780, A549, and PC3 cell lines in the dark. Significantly, the higher Pt accumulation well correlated with the higher photocytotoxicity of complexes **93** over **94**, indicating an enhanced positive effect of NO_2_ modification over NMe_2_ modification in 1,8-naphthalimide (Zhao et al., 2013).

In addition, the convenience of this type of axial ligand derivation has led to the synthesis of a series of dinuclear photoactive Pt(IV) anticancer prodrugs through bridging dicarboxylate ligands (**95–98**, Table 8) (Shi et al., 2018). The dinuclear complexes have IC_50_ values ranging from 8.8 to 78.3 μM toward A2780 human ovarian and esophageal OE19 cell lines while being nontoxic toward normal MRC5 cells. The di-nucleation of Pt(IV) center did not alter the photoactivation wavelength of the resulting Pt(IV) complexes in comparison to the mono-Pt(IV) counterparts. The dinuclear Pt(IV) complexes have good dark stability, but it can be activated by blue light at 420 nm accompanied with release of bridging ligand, mononuclear photolytic Pt(II) products, and generation of azidyl and hydroxyl radicals. The introduction of the bridging aromatic ligand in complex **97** significantly enhanced its cellular uptake which was more than five times higher than those of **95**, **96,** and **98** in all selected cell lines, attributable to its aromatic bridge linker with high lipophilicity, while complex **98** with relatively longer aliphatic linkers had the poorest ability to accumulate in cancer cells, but it showed more significant changes in the cell cycle progression and higher photocytotoxicity than **97**. This implicated that the cytotoxicity of these subgroups of Pt(IV) prodrugs is not simply and directly correlated with the cellular accumulation of them. Both **97** and **98** inhibited cell cycle progression from G2/M to G0/G1 upon irradiation. This was different to cisplatin which was showed to arrest cells at the S phase (Muhammad et al., 2017), suggesting a different mechanism of action of complexes **97** and **98** from cisplatin. Further study in the interaction of **97** and **98** with 5′-GMP showed that the Pt centers in **97** and **98** did not react with 5′-GMP until they were detached from the bridging ligands (Shi et al., 2018). However, all of the four dinuclear complexes are less active toward A2780 than the corresponding mononuclear complex **36**. This suggests that specific attention should be paid to design appropriate bridging ligands to promote simultaneously the cellular uptake and photoactivity of the resulting polynuclear Pt(IV) prodrugs.

## Remarks and Prospects

Due to highly spatiotemporal controllability, photoactivatable Pt(IV) complexes have emerged to be the most promising candidates of the Pt(IV) prodrug family and showed encouraging potential to conquer the high toxicity and resistance of clinic Pt(II) anticancer drugs. We focused on photoactivated Pt(IV) prodrugs with the general octahedral structure as [Pt^IV^(N_1_)(N_2_)(L_1_)(L_2_)(A_1_)(A_2_)], where N_1_ and N_2_ are non-leaving nitrogen donor ligands, L_1_ and L_2_ are leaving ligands, and A_1_ and A_2_ are axial ligands, to discuss the effects of diverse ligands on their photochemistry and photoactivity.

Among the leaving ligands, the nature of the planar ligand is directly related to the absorption of the LMCT bands, but it does not determine the excitation wavelength alone. The studies reported so far have demonstrated that azide is the best-leaving ligand, and the biological activity of *trans*-platinum is higher than that of *cis*-platinum. On the one hand, the ligand in the *trans* position is more favorable for electron transfer from the ligand to the metal center. On the other hand, the *trans*-platinum pattern is more likely to form multiple forms of cross-links (DNA–DNA, DNA–protein, and intra-DNA) in cells.

For non-leaving ligands, steric effects significantly affect the nature of platinum complexes, but the choice of the most suitable ligand is still being explored. Overall, the presence of conjugated ligands significantly enhances the cytotoxicity and photoactivity of platinum. The better example is complex **36**, which has a higher light-to-dark cytotoxicity index and longer exciting wavelength (blue or green light). Both of the steric hindrance and electronic effects of ligands on the platinum center of the ligands influence the physiological properties of the Pt(IV) prodrugs to a certain extent.

In the axial positions, the choice of different ligands brings versatile functions and significantly changes solubility, targeting ability, and pharmacological properties of Pt(IV) prodrugs. This makes the axial modification an excellent strategy, deserving further explorations (Xu et al., 2021a). Although the nature of the ligands and the length of the linker between ligands and Pt center in the axial positions remarkably affect the properties of Pt(IV) prodrugs, no apparent regularity has been found. The appropriate conjugation pattern and linker style also need further exploration. In recent years, a new strategy by combining PACT and PDT/PTT emerged, in which such combination may endow the Pt(IV) prodrugs’ good photocytotoxicity under higher excitation wavelength and higher potential to clinical applications.

In summary, coordination ligands significantly affect the photochemistry and photoactivity of anticancer Pt(IV) prodrugs. However, the process of photoactivation of Pt(IV) prodrugs in complex biological systems is still not fully understood, and the appropriate design of target Pt(IV) prodrugs remains a challenge. Although a few of the early developed Pt(IV) prodrugs, for example, ormaplatin, iproplatin, and satraplatin, have entered into clinical trials, and none of the photoactivatable Pt(IV) prodrugs developed later have entered this stage, meaning far way to go for this group of Pt(IV) prodrugs. Chemists can further design, synthesize, and test novel Pt(IV) prodrugs by focusing on three directions: i) extending activation light wavelength, ii) obtaining high photocytotoxicity and dark stability, and iii) gaining tumor-specific accumulation with promising druggability. Meanwhile, pharmacist and pharmacologists must also be engaged to deeply cooperate with chemists to promote the carrying out of more physiological tests and pre-clinical and even clinical tests.
